# Biomarkers for polycyclic aromatic hydrocarbons in human excreta: recent advances in analytical techniques—a review

**DOI:** 10.1007/s10653-023-01699-1

**Published:** 2023-08-02

**Authors:** Katarzyna Styszko, Justyna Pamuła, Agnieszka Pac, Elżbieta Sochacka-Tatara

**Affiliations:** 1 Department of Coal Chemistry and Environmental Sciences, Faculty of Energy and Fuels, AGH University of Science and Technology, al. Mickiewicza 30, 30-059 Kraków, Poland; 2 Department of Geoengineering and Water Management, Faculty of Environmental Engineering and Energy, Cracow University of Technology, Kraków, Poland; 3 Chair of Epidemiology and Preventive Medicine, Jagiellonian University Medical College, Kraków, Poland

**Keywords:** Polycyclic aromatic hydrocarbons, Metabolites, Human biomonitoring, Chromatographic analysis

## Abstract

Polycyclic aromatic hydrocarbons (PAHs) are widespread environmental pollutants that are generated by the incomplete combustion of organic materials. The main anthropogenic sources of PAHs are the combustion of solid fuels for heating purposes, illegal waste incineration, road transport and industries based on fossil fuels. PAHs can easily enter the body because they are present in all elements of the environment, including water, soil, air, and food. Due to their ubiquitous presence, PAHs, may exert a harmful effect on human health. Assessing PAH exposure through biomonitoring mostly involve techniques to measure the concentration of 1-hydroxypyrene in human urine. Nevertheless, through recent progress in analytical techniques, other common metabolites of PAHs in human biospecimens can be detected. A scientific literature search was conducted to determine which hydroxy derivatives of PAHs are markers of PAHs exposure and to reveal the leading sources of these compounds. Techniques for analyzing biological samples to identify OH-PAHs are also discussed. The most frequently determined OH-PAH in human urine is 1-hydroxypyrene, the concentration of which reaches up to a dozen ng/L in urine. Apart from this compound, the most frequently determined biomarkers were naphthalene and fluorene metabolites. The highest concentrations of 1- and 2-hydroxynaphthalene, as well as 2-hydroxyfluorene, are associated with occupational exposure and reach approximately 30 ng/L in urine. High molecular weight PAH metabolites have been identified in only a few studies. To date, PAH metabolites in feces have been analyzed only in animal models for PAH exposure. The most frequently used analytical method is HPLC-FLD. However, compared to liquid chromatography, the LOD for gas chromatography methods is at least one order of magnitude lower. The hydroxy derivatives naphthalene and fluorene may also serve as indicators of PAH exposure.

**Supplementary Information:**

The online version contains supplementary material available at 10.1007/s10653-023-01699-1.

## Introduction

Polycyclic aromatic hydrocarbons (PAHs) are widely distributed environmental pollutants and are generated from the incomplete combustion of organic materials or pyrolysis processes. PAHs are a group of organic compounds that consist of two or more aromatic rings in various structural configurations and are characterized by chemical stability, low volatility and solubility in water (de Oliveira Galvão et al., 2017; Gilbert & Viau, 1997). Low molecular weight PAHs (LMW PAHs) contain up to four rings, while high molecular weight PAHs (HMW PAHs) contain more than four rings (Viau et al., 1995). The main anthropogenic sources of PAHs are solid fuel combustion, industry using fossil fuels, wood burning, illegal waste burning and transport (Nethery et al., 2012). Natural sources include forest fires and volcanic eruptions, but this process generates relatively small amounts of PAHs compared to the amount of PAHs with anthropogenic origin (Xia et al., 2009). Individual exposure to PAHs occurs through the intake and preparation of grilled and smoked foods (Zhu et al., 2009). The ubiquitous presence of PAHs has been confirmed, inter alia, in sediments, soil, living organisms and the atmosphere. PAHs enter the human body mainly through the respiratory tract but also in water and soil, from which they enter the food chain and are consumed by humans through food (Elovaara et al., 2003). PAHs have been of interest in environmental research for years because some of these compounds are highly carcinogenic or mutagenic; additionally, they cause respiratory and cardiovascular diseases and contribute to the birth of premature or underweight children (Xu et al., 2004). Most high-molecular weight (HMW) PAHs slowly degrade and are therefore a potential long-term health hazard to humans and animals (Yang et al., 2014). In particular, benzo[a]pyrene (BaPYR), consisting of five rings, has been classified by the International Agency for Research on Cancer as a compound with carcinogenic activity (Fiala et al., 2001). Undoubtedly, the most dangerous effects caused by exposure to BaPYR include internal organ cancer, such as lung cancer (Hansen et al., 1993). According to the World Health Organization, outdoor air pollution is responsible for 3.4 million early deaths each year. Gaseous compounds and particulate matter are the main components of air pollutants. Both of them contain PAHs; however, HMW PAHs are predominantly present in the PM fraction. The effect of PM on health depends, among on the aerodynamic diameter of a PM particle, among other factors (Choi et al., 2006; Gruszecka-Kosowska, 2018; Gruszecka-Kosowska & Wdowin, 2016; Samek, 2016; Styszko et al., 2017; Zwozdziak et al., 2017). Small particles with diameters of less than 2.5 μm (PM2.5) are particularly dangerous because they may penetrate the lung alveoli and enter the bloodstream, thus exerting adverse health effects. That is why the United States Environmental Protection Agency (USEPA) has prepared a list of sixteen PAHs and issued a recommendation to control these PAHs in major environmental elements, such as water, soil, plants and air. Basic information on 16 PAHs provided by the USEPA is listed in Table S1.

In Poland and other European countries, the concentration and chemical composition of particles varies from season to season, as there are different sources of air pollutants (Rogula-Kozłowska et al., 2014; Samek et al., 2017). As a result, the exposure to PAHs varies in winter and summer. Stoves, boilers and furnaces with low energy efficiency are still being used in many individual households, which does not guarantee proper combustion conditions. An increase in BaPYR emissions from “low emissions” sources during the winter, combined with poorer conditions for the dispersion of pollutants in the cold season, contributes to an explicit increase in daily BaPYR concentrations during this particular period (Heudorf & Angerer, 2001a).

The European Environmental Agency (EEA) has been monitoring environmental pollution for years. Among the air pollutants mentioned above, BaPYR functions as a marker for the carcinogenic risk of PAHs in the environment (Van Wijnen et al., 1996). A 2020 report on air quality in Europe prepared for the European Union presents the average annual BaPYR concentrations for air pollution monitoring stations in Europe (data from 2018). The report includes Norway and Switzerland, as well as all EU Member States except Greece, Malta and Portugal (European Environment Agency (EEA) 2020). The average annual target for PM10 pollution is 1 ng/m^3^ (Official Journal of the European Union, 2005). Of the 25 countries reporting data on BaPYR, 14 showed concentrations higher than the target value. The data indicates that Poland had the greatest average annual concentrations of BaPYR in the EU. Other countries with high levels of air pollution by BaPYR include the Czech Republic, Slovakia and Bulgaria. However, it is crucial to consider the number of reporting stations in each countries in the Report. The number of monitoring stations per 1000 km^2^ is similar in the Czech Republic and Poland (0.47 and 0.44, respectively). For Bulgaria and Slovakia, this number is smaller (0.13 and 0.06, respectively). One monitoring station occurs in less than 5000 km^2^ for only eight countries (Belgium, Czech Republic, Italy, Poland, Luxembourg, Austria, Germany and Switzerland). Poland, the Czech Republic, Austria, Italy and Germany are among the 13 countries with an average BaPYR concentration above the target. Croatia is a notable example in the EEA ranking. There are only three measuring stations there, so each covers slightly less than 20,000 km^2^. The average annual BaPYR concentration oscillates approximately 2 ng/m^3^. It is recognized that the densification of the monitoring network would improve the quality of the data collected. In addition, air quality control could be enhanced and more precise results could be obtained, especially for countries in which a singular monitoring station covers a large area. In addition, the EEA summary does not include countries in southeastern Europe, such as North Macedonia, Turkey, Serbia, Montenegro, Bosnia and Herzegovina. These countries are characterized by a high concentration of PM, which is potentially associated with significant amounts of BaPYR in the air (Leroyer et al., 2010).

PAHs are absorbed in the body through the following routes: food, dermal exposure and inhalation (Göen et al., 1995). Among the air pollutants, particulate matter should be distinguished, especially PM2.5 and PM1 fractions, since they easily condense and absorb PAHs (Preuss et al., 2004). Particles of small size, along with PAHs, enter the alveoli through the inhaled air and then enter the bloodstream, causing detrimental effects (Shahsavani et al., 2017).

After entering the human body, PAHs undergo a multistage detoxification process. In the mixed function oxygenase system, phase I enzymes easily metabolize PAHs into more hydrophilic products (hydroxylated derivatives). Some of the PAHs can be excreted directly as unconjugated polar metabolites; however, most undergo the second phase, in which they are conjugated with sulfate or glucuronic acid to form compounds that are more water-soluble (Sochacka-Tatara et al., 2018). Through the second phase of the process, PAHs can be excreted in feces and urine within a few days (Mori et al., 2011). HMW PAHs are primarily excreted in feces and urine, while LMW PAHs are primarily excreted in urine (Toriba, 2018). In addition to biomonitoring, PAHs and their metabolites can be measured in biological samples to determine the level of exposure in humans. In comparison with other physiological fluids, urine is suitable because it is easy and noninvasive to obtain samples, and it is available in large quantities, so even very low concentrations of substances can be examined (Boogaard & van Sittert, 1995). Among the most common biomarkers for PAH exposure is 1-hydroxypyrene, a metabolite of pyrene, which is always present in mixtures of PAHs; therefore, the metabolite functions as an indicator for general PAH exposure as well as pyrene exposure (Van Delft et al., 2001).

The purpose of the study is to review literature that analyzes the metabolites of PAHs in urine and/or feces to monitor environmental PAH exposure; the specific goals are to identify which PAHs are most frequently hydroxylated and which analytical methods are most commonly applied.

## Literature search

Accessible literature was found through searching the two largest global scientific databases, Science Direct and Scopus, which are both published by Elsevier Publishing. The search spans when the publications were first indexed in these databases to May 2021. The oldest publication dates back to 1987, while the newest one is from 2021. The search covers the time period through May 2021 and is based on article titles, abstracts, and keyword matches. The following terms were taken into consideration: 1-hydroxypyrene, monohydroxy polycyclic aromatic hydrocarbons, biomarker of PAH exposure, PAH metabolites, human biomonitoring and urine and feces. This review included studies written in English that addressed the presence of OH-PAH in urine and/or feces.

## Metabolites of PAHs

**Fig. 1:**
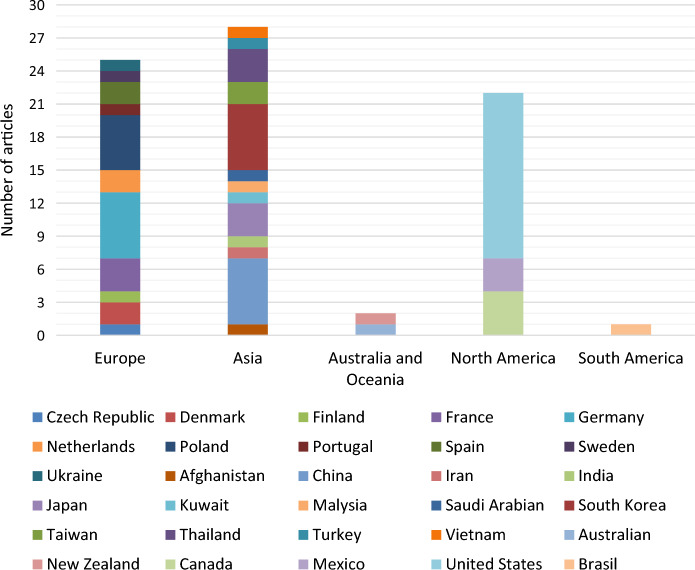
Number of scientific publications that analyzed the occurrence of OH-PAHs in urine per country since 1995

A total of 73 scientific publications from 28 countries were found. Figure 1 depicts the global distribution of articles that analyzed OH-WWA in urine, and the number of articles (case studies) per country was considered in this review. The majority of publications were from Asia (43%), followed by Europe (36%) and North, South America, Australia and Oceania (7%). The USA had the highest number of items per country, as shown in Fig. 1.

Among the articles reviewed, 37% examined PAH biomarkers in urine within children and adolescents, 58% investigated adults, and 5% involved children and adults. Over 40,000 urine samples were tested in total. Almost half of the tests cover less than 100 urine samples, more than 35% cover 100 to 1000 urine samples, and the rest cover 1000 to fewer than 6500 samples.

### PAH metabolites identified in urine

The concentration of monohydroxy metabolites PAHs in urine has been assessed in individuals worldwide. Table S2 summarizes the concentrations of various OH-PAHs for people worldwide as uncorrected (ng/mL urine) and creatinine corrected (µg/g creatinine) data (Tables S3a and b.). In addition, the country, research area, source of PAHs, and population size with age are provided in each table.

#### 1-Hydroxypyrene

**Fig. 2:**
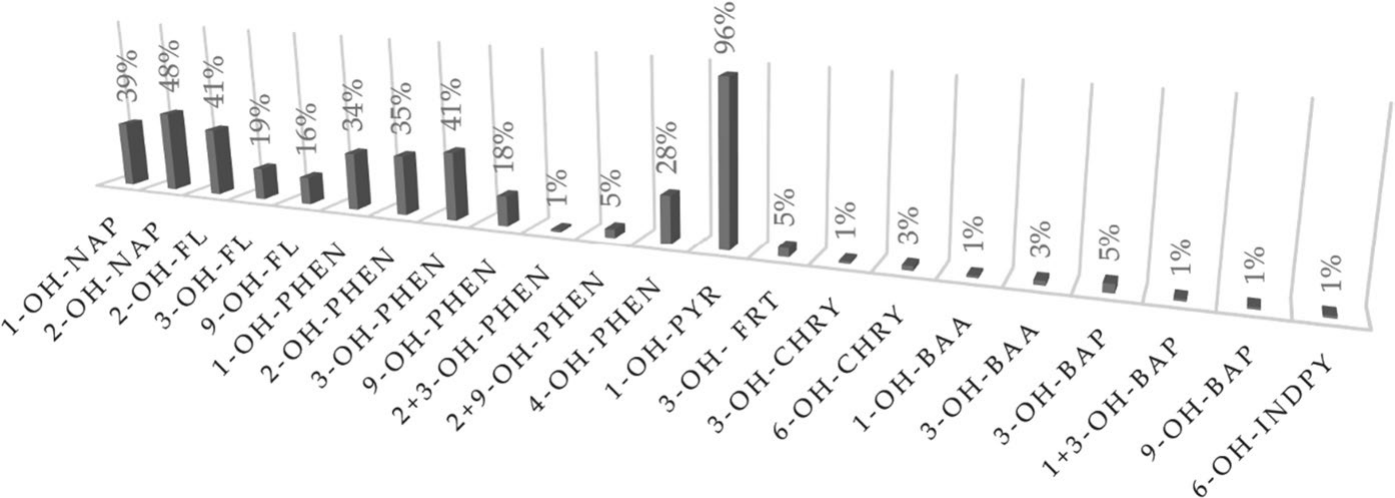
Frequency by which individual compounds have been identified in the analyzed publications

The most commonly used PAH metabolite in the form of hydroxy derivatives found in human urine is 1-hydroxypyrene. The compound has been used to assess occupational exposure to PAHs since the 1980s, and a decade later, it was used to assess environmental exposure to PAHs; this is because the compound can be feasibly identified at low levels (Cavanagh et al., 2007). In addition, the compound appeared in 96% of all analyzed publications, confirming its prominence (Fig. 2).

An article prepared by a group of scientists led by Hansen et al., (2008) contains detailed characteristics of 1-OH-PYR in environmental and occupational studies. To avoid duplicating the findings presented in that article, the remaining OH-PAHs were focused on. In brief, the lowest observed concentrations of 1-OH-PYR are 0.0742 ng/mL urine (geometric mean), the source of which is environmental tobacco smoke ETS, diet, and traffic density (Ochoa-Martínez et al., 2017), and creatinine at 0.037 to 0.0083 µg/g (median), the source of which is air pollution (Martínez-Salinas et al., 2010). Employees at a coke oven facility had the highest median and mean values, respectively, at 15.4 ng/mL urine (Ochoa-Martinez et al., 2016) and 16.361 µg/g creatinine (Sochacka-Tatara et al., 2018). Other occupations associated with high concentrations of 1-PYR-OH in urine include carbon electrode manufacturing, impregnation of road stones, aluminum smelting, glass manufacturing, chimney-sweeping, meat smoking, municipal and industrial waste incineration, metallurgical and petrochemical industries, and work in engine repair shops (Alghamdi et al., 2015; Mielzyńska et al., 2006; Oliveira et al., 2016; Siwińska et al., 1999).

#### Other OH-PAHs

In addition to 1-OH-PYR, various PAH metabolites are detected in urine. These metabolites are typically naphthalene hydroxy compounds, and every fourth publication includes these compounds (Fig. 2). Compared to 1-OH-PYR, the concentration of 1- and 2-OH-NAP in urine are almost a hundred times greater. Similar to 1-OH-PYR (Lee et al., 2001), samples from coke oven employees have the greatest concentrations of OH-NAPs: 1-OH-NAP 25.107 and 2-OH-NAP 30.078 (Xu et al., 2004), in ng/mL (median) 1-OH-NAP 46.2 and 36.2 (Campo et al., 2010). Urine from workers in Finland’s asphalt paving industry who are exposed to hazardous asphalt fumes while working was examined. The study (Väänänen et al., 2003) found that 1-OH-NAP concentrations were high, ranging from 16.568 to 21.666 µg/g creatinine (geometric mean). Concentrations of 1-OH-NAP and 2-OH-NAP up to 18.864 and 25.706 µg/g creatinine were found in samples from farmers, taxi drivers, and traffic police officers, respectively, in Thailand (Chetiyanukornkul et al., 2006). The concentration of OH-NAPs in urine was nearly four times lower in the US personnel exposed to jet propulsion fuel inhalation (Rodrigues et al., 2014). The presence of OH-NAPs in the urine at a level of several µg/g creatinine also results from living in metropolitan areas and air pollution (Li et al., 2010; Preuss et al., 2004; Sochacka-Tatara et al., 2018). Compared to eating grilled food, inhaling polluted air leads to higher amounts of PH-NAPS in urine (Li et al., 2012). Whether a person has been exposed to PAHs from smoking can be inferred from the amount of OH-NAPs present in their urine. Urine from smokers contained 1-OH-NAP concentrations that were 12 times higher and 2-OH-NAP concentrations that were 6 times higher than those of nonsmokers (Meeker et al., 2007).

Fluorene is marked with 2-, 3-, and 9-OH-FL hydroxy derivatives, but 2-OH-FL is the most prevalent marker because the presence of 2-OH-FL in urine was mentioned in 40% of the papers considered (Fig. 2). Industrial settings are associated with higher OH-FL concentrations. Samples from employees of Polish coking plants contained median hydroxyfluorene concentrations of 27.6 ng/mL of urine for 2-OH-FL and 11.9 ng/mL of urine for 9-OH-FL (Campo et al., 2010). On the other hand, samples from Chinese workers at a coking plant contained 2-OH-FL concentrations of 38.015 µg/g creatinine (mean) (Xu et al., 2004). Additionally, the presence of fluorene metabolites in urine is associated with exposure to air pollutants and ambient cigarette smoke (Li et al., 2010; Liu et al., 2017; Nethery et al., 2012; Sochacka-Tatara et al., 2018; Thai et al., 2016; Xia et al., 2009) and urine fluorene metabolites (Chetiyanukornkul et al., 2004; Dobraca et al., 2018; Khoury et al., 2018; Wang et al., 2017). The amount of 2-OH-FL found in the urine of citizens from Krakow, Poland, and Nanjing, China, support that these individuals suffer from smog. The concentrations are 2.90 µg/g creatinine (geometric mean) (Xia et al., 2009) and 0.942 µg/g creatinine (Siwińska et al., 1999). However, people in Canada, in which steel manufacturing is a major source of air pollution, only have 0.216 µg/g of creatinine (geometric mean) in their urine (Nethery et al., 2012). Smokers have a 2-OH-FL concentration that is four times higher than that of nonsmokers (Chetiyanukornkul et al., 2004). Interestingly, fluorene metabolites in the urine of individuals who consume grilled or smoked meals can reach 12.2 µg/g of creatinine (Li et al., 2012).

Phenanthrene metabolites are another group of OH-PAHs that are often studied, and more than 40% of the papers covered 3-OH-PHEN (Fig. 2). Exposure to ETS is the main factor contributing to the presence of OH-PHENs in urine as a result of air pollution (Becker et al., 2002; Guo et al., 2013; Heudorf & Angerer, 2001a; Schulz et al., 2008; Wang et al., 2017). Parquet glue that contains coal tar is another air contaminant that contributes to the presence of phenanthrene metabolites in urine. Here, the amount of OH-PHENs in the urine is less than 0.5 µg/g creatinine and is equivalent to the results obtained for ETS exposure. Concentrations of OH-PHENs from eating grilled or smoked food are substantially higher than those resulting from smoking-related exposure. While the concentration of 4-OH-PHEN is slightly over 0.7, the amounts of 1, 2, and 3-OH-PHEN in urine are over 2 µg/g creatinine (mean) (Li et al., 2012). Additionally, the presence of OH-PHENs in urine is linked to occupational exposure. Samples from employees at coking plants contained mean 1-OH-PHEN and 3-OH-PHEN concentrations of 11 and 23 µg/g creatinine, respectively (Xu et al., 2004). The maximal concentration of OH-PHENs determined did not surpass 1 µg/g of creatinine for steel manufactures inhaling contaminated air (Nethery et al., 2012).

Indeno(1,2,3-c,d)pyrene, chrysene, benzo(a)anthracene, and benzo(a)pyrene are hydroxyl derivatives of fluoranthene, which is described in the articles. Up to 5% of publications have focused on these substances.

In these works, 3-hydroxyfluoranthene is the sole fluoranthene metabolite that was discussed. The concentration of this chemical in the urine of coking plant workers was 0.014 µg/g creatinine (mean), which is 11 times greater than for nonexposed individuals. Additionally, 1-OH-FRT was found in the urine of each group of workers, with the control group having a 1-in-7 prevalence (Xu et al., 2004). On the other hand, samples from Americans without occupational exposure to PAHs showed levels of this chemical comparable to those of Chinese coking factory workers (Grainger et al., 2006). However, the highest quantities of 3-OH-FRT was found in samples from individuals exposed to PAHs because their homes are close to busy streets or industrial sources or due to household dust, house dust, or soil contamination. The average concentration of the fluoranthene metabolite in adults was 0.135 µg/g creatinine, while it was one order of magnitude lower in children (Chuang et al., 1999).

The chrysene metabolites 3-OH-chrysene and 6-OH-chrysene have been identified in urine. These OH-PAHs are linked to exposure to polluted air in the coking industry through inhalation. The mean concentration of 3-OH-CHRY was 1.382 µg/g creatinine, while that of 6-OH-CHRY was 63 times less (Xu et al., 2004). Both adults and children contain 6-OH-CHRY in their urine as a result of nonwork-related inhalation. This molecule was present in children at 0.05 µg/g creatinine (mean), more than three times lower than in adults (Chuang et al., 1999).

In Sweden, urine samples from chimney sweeps were examined for the presence of certain OH-PAHs. According to these studies, the median concentration of 3-hydroxy-benzo(a)anthracene in urine is 6.28 ng/mL (Alhamdow et al., 2017). The presence of benzo(a)anthracene metabolites was also established in the aforementioned paper by Chuang et al. The highest levels of 3-OH-BaA were found in adults, in which the mean concentration was 0.346 µg/g creatinine (Chuang et al., 1999).

1, 3 and 9-OH-BaP are benzo(a)pyrene metabolites that can be detected in urine. The average 3-OH-BaP levels in urine from chimney sweeps exposed to soot were 4.75 ng/mL, whereas the highest levels were nearly 10 times higher (Xu et al., 2004). Samples from employees at coking plants contained levels of 9-OH-BaP in their urine that averaged 0.45 ng/mL, but 3-OH-BaP was 5 times less prevalent (Chuang et al., 1999). In investigations conducted in France, the amount of 3-OH-BaP in soak was two tenths of a nanogram per milliliter of urine maximum, regardless of the source of PAH exposure (including was industry, metallurgy, or smoking) (Barbeau et al., 2011; Lafontaine et al., 2006; Leroyer et al., 2010). The sole article that discussed the presence of 1-OH-BaP in urine was written under Chuang’s supervision. The results are given as the sum of the 1-OH-BaP and the 3-OH-BaP in the urine. These analytes were detected in children at a level of 0.06 ng/mL urine, which is one hundredth higher than that in adults. According to these studies, a number of factors related to poor family income may be the source of PAHs (Chuang et al., 1999).

The same publication also revealed that the subject’s urine contained 6-hydroxy indeno(1,2,3-c, d)pyrene. The concentration of this substance in samples from children was 0.034 µg/g creatinine (mean), which was twice as high as the concentration in adults (Chuang et al., 1999).

### Determined PAH metabolites in feces

No report has examined PAH metabolites in human feces. To date, only animal models have been studied to investigate the excretion of PAH metabolites in excrement. Rat feces were examined by the van Schooten-led team for the presence of 1-OH-PYR and 3-OH-BaP. Urine and feces from exposed animals have been found to contain these two metabolites. Notably, metabolites were found at substantially higher concentrations than the original substances following the administration of the PAHs. BaPYR was only 9% of the initial dose that was eliminated as 3-OH-BaPYR in the urine and feces. Only 0.2% of the initial dose of the pyrene metabolite is excreted in the urine, compared to 17% in feces (Van Schooten et al., 1997). BaPYR was administered orally to rats in additional trials, and the animals’ feces were subsequently examined. Animals excreted approximately 72% of the total dose of administered drug, and metabolite excretion was three times greater in feces than urine (Moreau et al., 2015; van de Wiel et al., 1993). Twenty-four hours after administration, 45% of the dosage of BaPYR was eliminated in feces and urine, according to research by Ramesh et al. (Ramesh et al., 2001). The amount of OH-BaPYR excreted in rat excrement increased gradually from 4 to 72 h after exposure (Ramesh et al., 2002). The percentage of 1-OH-PYR excretion was much lower than that of 3-OH-BaPYR. Following intravenous administration, the mean cumulative percent of dosage excreted as 1-OH-PYR in urine over 24 h ranged from 1.7 to 3.2%, while biliary values ranged from 6.5 to 9.5% (Bouchard & Viau, 1998; Bouchard et al., 1998). In trials conducted by Marie et al., in which approximately 60% of the 24-h 1-OH-PYR was collected within the initial 0–8 h period (Marie et al., 2010), far higher values were reported. According to the groundbreaking animal experiments mentioned above, HMW PAH metabolites are eliminated more frequently in feces than in urine. Unfortunately, only two compounds were available for comparison (1-OH-PYR and 3-OH-BaPYR) because there were so few literature items.

## Techniques for analyzing biological samples to identify OH-PAHs

TablesS4a and b provides a summary of journal papers published over the last thirty years on the detection of OH-PAHs in urine samples. To the best of the authors’ knowledge, each chemical described was assigned a set of analysis techniques, detection limits, quantification limits, recovery values, and a description of the solid phase extraction technique that was employed. Gas chromatography combined with tandem mass spectrometry or liquid coupled with fluorescence detection and tandem mass spectrometry were used to detect the presence of OH-PAHs in urine. These procedures are based on approaches used to selectively separate chemical components from complicated mixtures.

**Fig. 3:**
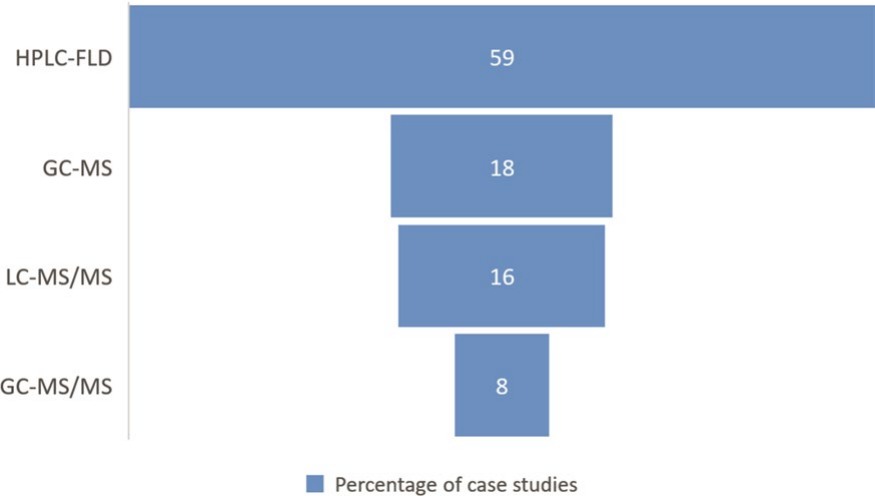
Percentage of each analytical method used in the analyzed case studies

Based on the acquired statistics, the most popular technique involves applying high-performance liquid chromatography with a fluorescence detector, which was first used by Jongeneelen to conduct ground-breaking research (Jongeneelen et al., 1987). Nearly, 60% of the scientific articles examined used the HPLC-FLD analytical technique (Fig. 3). Three substances—1-hydroxypyrene, 3-hydroxybenz(a)anthracene and 3-hydroxybenzo(a)pyrene—were the initial targets of this methodology. The application of HLPC-FLD to investigate nine more compounds with up to four benzene rings was expanded by Chetiyanukornkul et al., (2006). HPLC-FLD is used in many countries on five continents, including Europe (Czech Republic (Fiala et al., 2001), Denmark (Hansen et al., 2006; Van Wijnen et al., 1996), Finland (Väänänen et al., 2003), France (Barbeau et al., 2011; Lafontaine et al., 2006; Leroyer et al., 2010), Germany (2001b; Göen et al., 1995; Heudorf & Angerer, 2001a; Preuss et al., 2004), Portugal (Oliveira et al., 2016), Poland (Mielzyńska et al., 2006; Siwińska et al., 1998, 1999), Spain (Freire et al., 2009), Ukraine (Mucha et al., 2006), Asia (Afghanistan (Hemat et al., 2012)), China (Yang et al., 2018), Iran (Shahsavani et al., 2017), Japan (Mori et al., 2011; Toriba, 2018), Saudi Arabian (Alghamdi et al., 2015), South Korea (Alghamdi et al., 2015; Sul et al., 2012; Yoon et al., 2012), Taiwan (Kuo et al., 2004; Tsai et al., 2003), Thailand (Chetiyanukornkul et al., 2004, 2006; Naksen et al., 2017), North America, (Canada (Gilbert & Viau, 1997)), Mexico (Martínez-Salinas et al., 2010; Ochoa-Martinez et al., 2016; Ochoa-Martínez et al., 2017), USA (McClean et al., 2004), South America (Brasil (de Oliveira Galvão et al., 2017)), Australia and Oceania (New Zealand (Cavanagh et al., 2007)). To prepare a sample for analysis, urine is adjusted to pH 5 and is undergoes enzymatic hydrolysis with *β*-glucuronidase/aryl sulfatase; then, the sample is incubated for a specified period of time at constant temperature (Li et al., 2006) and the SPE process is performed to select analytes using SPE C18 cartridges of the Sep-Pak (Jongeneelen et al., 1987; McClean et al., 2004; Mori et al., 2011), the Pol-Sep (Siwińska et al., 1998, 1999), the VertiPak (Naksen et al., 2017), the Bond Elut (Väänänen et al., 2003) or ASPEC system (Barbeau et al., 2011). The SPE recoveries are summarized in Tables S4a and b. The volume of urine needed for each analysis ranges from 0.4 mL(Shahsavani et al., 2017) to 20 mL (Siwińska et al., 1998), but 10 mL (Freire et al., 2009; Naksen et al., 2017; Sul et al., 2012; Yoon et al., 2012) is the most typical amount. Enzymatic hydrolysis requires 4 µL (de Oliveira Galvão et al., 2017) to 100 µL (Freire et al., 2009; Llop et al., 2008) of reagent. The incubation time also varies and ranges from 1 h (Lafontaine et al., 2006) to as much as 20 h (Cavanagh et al., 2007), but the most popular is several hours (Kang et al., 2002; Martínez-Salinas et al., 2010; Naksen et al., 2017; Sul et al., 2012). The incubation temperature remains constant regardless of the sample preparation method utilized. In every study that was examined, 37 °C was applied.

In addition to HPLC-FLD, liquid chromatography and tandem mass spectrometry (LC‒MS/MS) are employed to determine the presence of OH-PAH in urine. Less than 20% of the publications used LC‒MS/MS (Fig. 3). Through this approach, OH-PAHs with up to five benzene rings can be identified; thus, this approach is quick, sensitive, selective, and reasonably all-encompassing (Alhamdow et al., 2017; Xia et al., 2009; Xu et al., 2004). The procedure used to prepare samples for LC‒MS analysis might be changed to exclude the solid-phase extraction stage step (Liu et al., 2017) or performed the same way as for HPLC-FLD (Xia et al., 2009; Xu et al., 2004). The sample can be mixed with n-hexane before centrifugation and freeze dried. Then, the dry residue can be dissolved in methanol to replace the SPE stage. The volume of urine needed for this analysis was 1 mL.

In the past 20 years, OH-PAHs in urine have also been identified using gas chromatography (GC). A total of 26% of the papers discussed used tandem mass spectrometry (GC‒MS/MS) or GC analytical procedures coupled to mass spectrometry (GC‒MS) (Fig. 3). Due to its ability to identify LMW and HMW PAHs, GC, particularly GC‒MS, offers a wide range of applications (Campo et al., 2010; Choi et al., 2017; Chuang et al., 1999; Li et al., 2012; Yang et al., 2014). Regardless of the detector utilized, sample preparation for GC analysis is a time-consuming and laborious process. The stages of hydration and incubation used for LC are necessary, then the samples are concentrated through extracting liquid samples. The extract is derivatized with a silylation reagent in the last stage. Solid-phase extraction (SPE) or liquid–liquid extraction is used to concentrate the sample (LLE). Pentane (2 × 5 mL)(Li et al., 2010) is often used for LLE(Rodrigues et al., 2014; Sochacka-Tatara et al., 2018; Thai et al., 2016). SPE is performed using C18 SPE cartridges (Campo et al., 2008) or EnvirElut PAH SPE cartridges (Grainger et al., 2006). The SPE recoveries are summarized in Tables S4a and b. The following reagents were used for derivatization: BSTFA [N,O-Bis(trimethylsilyl)trifluoroacetammide with 1% trimethylchlorosilane] or MSTFA [N-methyl-N-(trimethylsilyl)-trifluoroacetamide] (Schummer et al., 2009). The sample was incubated for 45 min at 90 °C using the Campo et al. approach, and 100 µL of BSTFA additive was used (Campo et al., 2008). For MSTFA, a variety of combinations can be used, including adding 50 µL of reagent and performing an hour-long incubation period at 60 °C (Wang et al., 2012), as well as the doubling of both parameters while maintaining the same temperature (Gaudreau et al., 2016). The most popular combination, however, is adding 10 µl of derivatives and performing a 30-min incubation at 60 °C (CDC Environmental Health, 2013; Li et al., 2006; Romanoff et al., 2006). Researchers from the Schummer-led team compared the efficacy of the two derivatives in their investigation. The optimal derivatization conditions for OH-PAHs were 20 min for MTBSTFA and 40 min for BSTFA, both at 60 °C. For derivatization, 30 µl of reagent was added. Due to the greater analytical responses of MTBSTFA, the authors recommend using this reagent (Schummer et al., 2009).

The most crucial stage in sample preparation, regardless of whether liquid or gas chromatography is utilized, is the separation of analytes from urine and the subsequent concentration of analytes. TablesS4a and b provide details on the applied solid phase extraction techniques found throughout the literature review. The best recovery values (over 80%) are shown by SUPELCO SPE cartridges, although they can only be used with 1,2-OH-NAP, 2-OH-FL, and 1-OH-PYR (Zhu et al., 2009). The best carbides are C18. This is because these extraction columns are more widely used and reduce analyte loss compared to Oasis WAX (Wang et al., 2017) or EnvirElut cartridges (Grainger et al., 2006). Most of the OH-PAHs that have been discussed have recovery values in the literature, and they start at 50% (Campo et al., 2008). Cartridge C18 is used to extract chemicals from aqueous solutions in trace levels, including those with weak hydrophobicity properties (Mcdonald & Bouvier, 2001) and are packed with silica-based sorbents.

In contrast to liquid chromatography, gas chromatography can be used to detect OH-PAHs in urine with lower concentrations, regardless of whether they are LMW or HMW PAHs; this was confirmed by the LOD values listed in Table S4bS4b. With this procedure, 27 of the 28 compounds that were mentioned can be identified, or approximately 100% of the OH-PAHs that are routinely examined in urine. 9-OH-BaPYR is the only hydroxy derivative of PAH that cannot be identified by gas chromatography. Except for 3-OH-BaPYR, all detection limits for gas chromatography were lower than those for liquid chromatography. The LOD for this chemical using HPLC-FLD is 325 times lower than that using GC‒MS (Leroyer et al., 2010; Li et al., 2006). However, regardless of whether GC (Khoury et al., 2018) or LC (Alhamdow et al., 2017) is employed, the LOD for coupled techniques using tandem mass spectrometry is the same (2 ng/L). With the exception of GC‒MS/MS, all analytical techniques indicate LODs ranging from 10^–3^ to 10^–1^ µg/L for 1-OH-PYR, the most frequently measured metabolite of urine PAH. LOD values for GC‒MS/MS range from 10^–3^ to 10^–2^ µg/L. In general, compared to liquid chromatography, the LOD for gas chromatography methods is at least one order of magnitude lower (see Table S4bS4b).

## Conclusion

The general population can be exposed to PAHs through air, water, soil, and food. Routes of exposure include ingestion, inhalation, and dermal contact in occupational and nonoccupational settings. Some exposures may involve more than one route simultaneously, affecting the total absorbed dose (such as dermal and inhalation exposures from contaminated air). Moreover, exposure involves a complex mixture of different PAHs. Once PAHs enter the human body, they are rapidly metabolized and leave the body within a few days, primarily through excretion in feces and urine. More specifically, low molecular weight PAH metabolites are primarily excreted in urine, whereas those with higher molecular mass are eliminated mainly via the bile with feces. Furthermore, excretion in the form of conjugates should be considered. Some PAHs can be excreted directly as unconjugated polar metabolites, but most undergo second phase conjugation with sulfate or glucuronic acid to form compounds with better water solubility.

To overcome difficulties in monitoring PAH exposure, human biomonitoring can be used. Biomonitoring techniques to identify PAH exposure involve measuring PAHs and their metabolites in blood and urine, measuring mutagenicity in urine and feces, and performing DNA and protein adduct formation. Urine is the preferred human biological matrix since it is noninvasive and easy to collect, and it is also accessible in large volumes; thus, in contrast to other fluids, very low concentrations of chemicals can be measured in urine. Numerous biomonitoring studies have reported a strict correlation between exposure to PAHs and the occurrence of their hydroxylated metabolites, especially 1-hydroxypyrene (1-OH-PYR), in human urine. Numerous studies have confirmed the correlation, but several other hydroxylated metabolites have also been proposed for makers to analyze urine samples and PAH exposure. To identify various sources of PAH exposure and achieve more precise exposure estimations, biomonitoring of PAH exposure should include other hydroxylated metabolites of PAHs, such as 2-hydroxynaphthalene (2-OH-NAP) and 2-hydroxyfluorene (2-OH-FLU), in addition to 1-OH-PYR. According to the literature that was gathered, the chromatographic methods used to determine the presence of OH-PAHs in urine are covered in great detail. Ultimately, it is important to acknowledge the advantages of HPLC-FLD over alternative analytical methods connected to mass spectrometers. This is a result of the method’s popularity, which can be attributed to the equipment’s accessibility, the quick sample preparation steps, and the method’s suitability for identifying LMW PAHs that are discharged in urine. Despite the possibility of superior chromatographic separation and a high level of specificity offered by gas chromatography, it is less popular due to the expensive and uncommon equipment, such as mass spectrometers.

Identifying OH-PAHs in urine is not difficult with the analytical techniques available. Due to the limited number of literature publications, research on feces remains in its early stages. This topic is niche, making it a potential area for scientific advancement. Moreover, very little information is available on the presence, distribution, and behavior of OH-PAHs in the environment. Although OH-PAHs have not been studied as extensively as PAHs, N-PAHs and O-PAHs, some studies have shown the potential of these compounds to induce toxicity and carcinogenicity. A common feature of the structure of estrogenic compounds is a phenol group with a hydrophobic moiety at the para position and no bulky group at the ortho-position. Therefore, the structural similarity of several OHPAHs to 17-estradiol induces the potency of estrogenic or antiestrogenic activities. The available data generally refer to OH-PAHs in tissues and fluids collected from aquatic and terrestrial organisms to be used as biomarkers for PAH contamination. Exposure monitoring programs to track chemical concentrations over space and time are a priority—these programs would provide important context for on-going biomonitoring programs and facilitate the design and implementation of health issues. Therefore, knowledge on PAH exposure is essential to establish policies for reducing emissions.

### Supplementary Information

Below is the link to the electronic supplementary material.

Supplementary file1 (DOCX 231 kb)

## Abbreviations

1-OH-NAP: 1-Hydroxynaphthalene
2-OH-NAP: 2-Hydroxynaphthalene
2-OH-FL: 2-Hydroxyfluorene
3-OH-FL: 3-Hydroxyfluorene
9-OH-FL: 9-Hydroxyfluorene
1-OH-PHEN: 1-Hydroxyphenanthrene
2-OH-PHEN: 2-Hydroxyphenanthrene
3-OH-PHEN: 3-Hydroxyphenanthrene
4-OH-PHEN: 4-Hydroxyphenanthrene
9-OH-PHEN: 9-Hydroxyphenanthrene
1-OH-PYR: 1-Hydroxypyrene
3-OH-FRT: 3-Hydroxyfluoranthene
3-OH-CHRY: 3-Hydroxychrysene
6-OH-CHRY: 6-Hydroxychrysene
1-OH-BaA: 1-Hydroxybenzo(a)anthracene
3-OH-BaA: 3-Hydroxybenzo(a)anthracene
1-OH-BaPYR: 1-Hydroxybenzo(a)pyrene
3-OH-BaPYR: 3-Hydroxybenzo(a)pyrene
9-OH-BaPYR: 9-Hydroxybenzo(a)pyrene
6-OH-IndPy: 6-HydroxyIndeno(1,2,3-c,d)pyrene
HPLC-FLD: High-performance liquid chromatography with fluorescence detection
LC‒MS/MS: Liquid chromatography–tandem mass spectrometry
GC‒MS: Gas chromatography‒mass spectrometry
GC‒MS/MS: Gas chromatography–tandem mass spectrometry
