# Supplementary material for: Biomarkers for polycyclic aromatic hydrocarbons in human excreta: recent advances in analytical techniques—a review
Source: Environ Geochem Health. 2023 Aug 2;45(10):7099–113. doi: 10.1007/s10653-023-01699-1 (PMC10517897; doi:10.1007/s10653-023-01699-1)
Supplement: Supplementary file 1 — Supplementary file1 (DOCX 231 kb) [file 10653_2023_1699_MOESM1_ESM.docx]

**Biomarkers for polycyclic aromatic hydrocarbons in human excreta: recent advances in analytical techniques—a review**

Katarzyna Styszko^1^, Justyna Pamuła^2^, Agnieszka Pac^3^, Elżbieta Sochacka-Tatara^4^

^1^Department of Coal Chemistry and Environmental Sciences, Faculty of Energy and Fuels, AGH University of Science and Technology, al. Mickiewicza 30, 30-059 Kraków, Poland, https://orcid.org/0000-0003-0092-3772

^2^Department of Geoengineering and Water Management, Faculty of Environmental Engineering and Energy, Cracow University of Technology, Kraków, Poland, https://orcid.org/0000-0001-7129-9562

^3^Chair of Epidemiology and Preventive Medicine, Jagiellonian University Medical College, Kraków, Poland, https://orcid.org/0000-0002-6057-479X

^4^Chair of Epidemiology and Preventive Medicine, Jagiellonian University Medical College, Kraków, Poland, https://orcid.org/0000-0002-2619-1947

Correspondence to Katarzyna Styszko (email: styszko@agh.edu.pl)

Supplementary Information

**Abbreviations**

1-OH-NAP – 1-hydroxynaphthalene

2-OH-NAP – 2-hydroxynaphthalene

2-OH-FL – 2-hydroxyfluorene

3-OH-FL – 3-hydroxyfluorene

9-OH-FL – 9-hydroxyfluorene

1-OH-PHEN - 1-hydroxyphenanthrene

2-OH-PHEN - 2-hydroxyphenanthrene

3-OH-PHEN - 3-hydroxyphenanthrene

4-OH-PHEN – 4-hydroxyphenanthrene

9-OH-PHEN – 9-hydroxyphenanthrene

1-OH-PYR - 1-hydroxypyrene

3-OH-FRT - 3-hydroxyfluoranthene

3-OH-CHRY – 3-hydroxychrysene

6-OH-CHRY – 6-hydroxychrysene

1-OH-BaA – 1- hydroxybenzo(a)anthracene

3-OH-BaA – 3- hydroxybenzo(a)anthracene

1-OH-BaPYR – 1- hydroxybenzo(a)pyrene

3-OH-BaPYR – 3- hydroxybenzo(a)pyrene

9-OH-BaPYR – 9- hydroxybenzo(a)pyrene

6-OH-IndPy – 6-hydroxyIndeno(1,2,3-c,d)pyrene

HPLC-FLD - high-performance liquid chromatography with fluorescence detection

LC-MS/MS - liquid chromatography - tandem mass spectrometry

GC-MS - gas chromatography-mass spectrometry

GC-MS/MS - gas chromatography - tandem mass spectrometry

ND - not detectable

NS - no specific

ME – median

GE - geometric mean

IQR - interquartile range

SE – standard error

CI - confidence interval

$\bar{x}$– arithmetic mean

SD – standard deviation

Table S1. The basic characteristics of 16 PAHs indicated by USEPA

|  | Name of compounds | Formula | CAS No. | Water solubility (mg/l) | Molecular weight (g/mol) | Number of rings | logP | Boiling point (°C) |
| --- | --- | --- | --- | --- | --- | --- | --- | --- |
| Low Molecular Weight PAHs | Naphthalene | 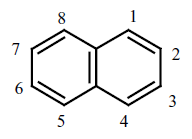 | 91-20-3 | 31 | 128.17 | 2 | 3.33 | 218 |
|  | Acenaphthylene | 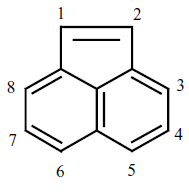 | 208-96-8 | 16.1 | 152.19 | 3 | 3.94 | 265 |
|  | Acenaphthene | 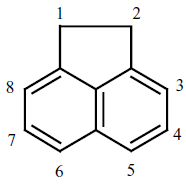 | 83-32-9 | 3.90 | 154.21 | 3 | 3.92 | 279 |
|  | Fluorene | 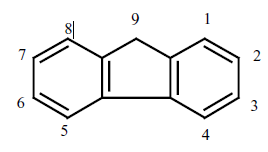 | 84987-80-4 | 1.69 | 166.22 | 3 | 4.18 | 295 |
|  | Phenanthrene | 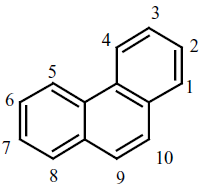 | 85-01-8 | 1.10 | 178.23 | 3 | 4.46 | 340 |
|  | Anthracene | 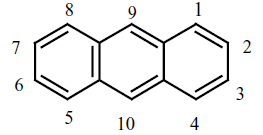 | 120-12-7 | 0.043 | 178.23 | 3 | 4.45 | 340 |
|  | Pyrene | 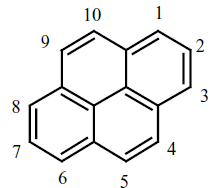 | 129-00-0 | 0.135 | 202.25 | 4 | 4.88 | 404 |
|  | Fluoranthene | 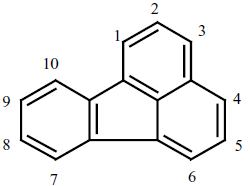 | 206-44-0 | 0.20 | 202.25 | 4 | 5.16 | 384 |
|  | Chrysene | 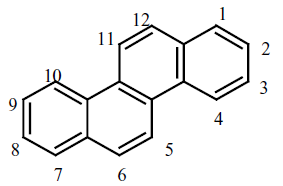 | 218-01-9 | 0.002 | 228.3 | 4 | 5.81 | 448 |
|  | Benz[a]anthracene | 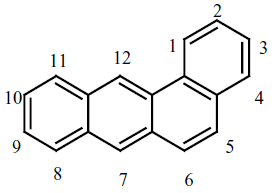 | 56-55-3 | 0,009 | 228.3 | 4 | 5.76 | 438 |
| High Molecular Weight PAHs | Benzo[b]fluoranthene | 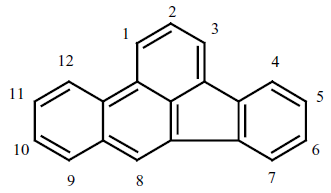 | 205-99-2 | 0.0015 | 252.3 | 5 | 6.12 | 481 |
|  | Benzo[k]fluoranthene | 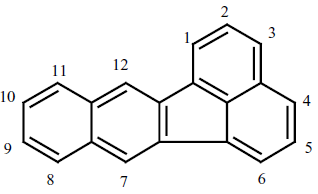 | 207-08-9 | 0.001 | 252.3 | 5 | 6.11 | 480 |
|  | Benzo[a]pyrene | 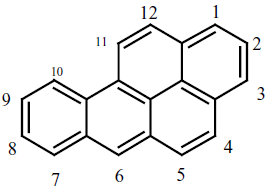 | 50-32-8 | 0.002 | 252.3 | 5 | 6.13 | 438 |
|  | Indeno[1,2,3-cd]pyrene | 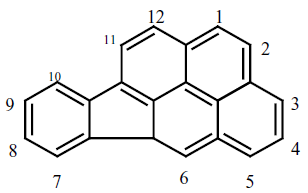 | 193-39-5 | 0.0007 | 276.3 | 6 | 6.58 | 536 |
|  | Benzo[ghi]perylene | 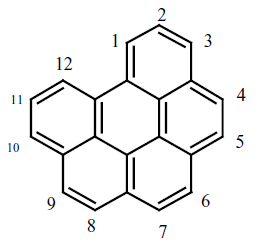 | 191-24-2 | 0.00026 | 276.3 | 6 | 6.63 | 550 |
|  | Dibenz[a,h]anthracene | 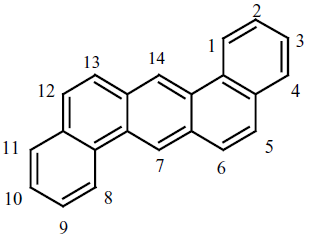 | 53-70-3 | 0.00249 | 278.3 | 6 | 6.75 | 524 |

Table S2. Review of urinary concentrations of OH-PAHs expressed in ng/mL depending on the country of determination

| **country** | **location** | **source of exposure** | **study population** | **age**  $\bar{x}$**±SD** | **statistic** | **OH-PAHs concentration** | | | | | | | | | | | | | |  |  |
| --- | --- | --- | --- | --- | --- | --- | --- | --- | --- | --- | --- | --- | --- | --- | --- | --- | --- | --- | --- | --- | --- |
|  |  |  |  |  |  | **1-OH-NAP** | **2-OH-NAP** | **2-OH-FL** | **3-OH-FL** | **9-OH-FL** | **1-OH-PHEN** | **9-OH-PHEN** | **2-OH-PHEN** | **3-OH-PHEN** | **4-OH-PHEN** | **1-OH-PYR** | **6-OH-CHRY** | **3-OH-BaA** | **3-OH-BaP** | **Analytical method** | **Reference** |
|  |  |  |  |  |  | **uncorrected (ng/ml urine)** | | | | | | | | | | | | | |  |  |
| Afghanistan | Kabul and rural area | air pollution - type of heating fuel used (wood and dung, diesel generators) and smoking | 55 | 5.5±2.3 and  31.6±8.6 | ME  (range) |  |  |  |  |  | 1.31  (0.0466-6.865) | sum of 2-OH-PHEN and 9-OH-PHEN | | 1.147  (0.0193-8.925) | 0.119  (0.004-0.976) | 1.646  (0.0714-16.288) |  |  |  | HPLC-FLD | (Hemat et al. 2012) |
|  |  |  |  |  |  |  |  |  |  |  |  | 0.783  (0.0463-2.903) | |  |  |  |  |  |  |  |  |
| Australian | Queensland | air pollution | 100 | between 0 and ≥60 | GM  (range) | 9.221  (0.924-375.182) | 4.104  (2.017-8.104) | 0.261  (0.095-0.634) | 0.132  (0.046-0.374) | 0.299  (0.135-0.57) | 0.134  (0.079-0.278) |  | 0.06  (0.024-0.13) | 0.081  (0.042-0.151) | 0.03  (0.012-0.065) | 0.142  (0.05-0.307) |  |  |  | GC-MS/MS | (Thai et al. 2016) |
| Canada | national research | environmental tobacco smoke ETS | 1975 | between 3 and 79 | 95th percentile  (CI) | 4.3  (2.7–5.9) | from 14 to 22  (8.2–29) | 0.71  (0.61–0.82) | 0.29  (0.19–0.38) | from 0.3 to 0.49  (0.22–0.6) | from 0.36 to 0.73  (0.28–1.0) | 0.098 (0.066–0.13) | from 0.098 to 0.18  (0.082–0.23) | 0.25 (0.19–0.32) | from 0.063 to 0.094  (0.051–0.14) | 0.27  (0.22–0.32) |  |  |  | GC-MS/MS | (Khoury et al. 2018) |
| China | Guangzhou, Harbin, Shahghain | Multiple (no identified industries) | 84 | 29± 16 | $\bar{x}$  ME  (95%CI) | 1.58  0.528  (0.621–2.54) | 6.96  2.27  (3.0-10.91) | 1.48  0.893  (1.06–1.9) |  |  |  | 0.351  0.217  (0.243-0.459) | 0.581  0.323  (0.366-0.796) | 0.714  0.387  (0.461-0.967) | 0.083  0.033  (0.016-0.151) | 0.667  0.378  (0.458-0.877) |  |  |  | LC-MS/MS | (Y. Guo et al. 2013) |
| China | Chongqing | air pollution | 1207 | between 7 and 13 | $\bar{x}$ ±SD  GM |  | 4.92±8.98  2.571 | 1.556±2.995  0.837 |  |  |  | 4.513±4.698  2.549 |  |  |  | 0.278±0.227  0.184 |  |  |  | LC-MS/MS | (Liu et al. 2017) |
| Germany | national research | environmental tobacco smoke ETS | 573 | between 18 and 69 | GM  (95%CI) |  |  |  |  |  | 0.38  (0.36-0.41) | sum of 2-OH-PHEN and 9-OH-PHEN | | 0.29  (0.27-0.32) |  | 0.13  (0.12-0.15) |  |  |  | HPLC-FLD | (Becker et al. 2002) |
|  |  |  |  |  |  |  |  |  |  |  |  | 0.24  (0.22-0.25) | |  |  |  |  |  |  |  |  |
| Germany | national research | environmental tobacco smoke ETS | 599 | between 3 and 14 | GM  (95%CI) |  |  |  |  |  | 0.185  (0.175-0.196) | 0.119  (0.113-0.126) | | 0.162  (0.153-0.172) |  | 0.129  (0.121-0.138) |  |  |  | HPLC-FLD | (Schulz et al. 2008) |
| India | Chennai, Mettuplayam | Multiple (no identified industries) | 3820 | 37±17 | $\bar{x}$  ME  (95%CI) | 3.39  1.11  (1.81-4.97) | 8.71  3.78  (5.07-12.36) | 1.11  0.346  (5.21-1.71) |  |  |  | 0.284  0.154  (0.147-0.421) | 0.388  0.256  (0.213-0.563) | 0.419  0.289  (0.284-0.554) | 0.054  0.027  (0.03-0.079) | 0.699  0.424  (0.451-0.948) |  |  |  | LC-MS/MS | (Y. Guo et al. 2013) |
| Japan | Ehime, Kumamoto | Multiple (no identified industries) | 34 | 32±10 | $\bar{x}$  ME  (95%CI) | 0.55  0.266  (0.219-0.81) | 5.29  3.25  (1.72-8.87) | 0.22  0.207  (0.164-0.276) |  |  |  | 0.086  0.077  (0.064–0.109) | 0.099  0.065  (0.053–0.145) | 0.151  0.121  (0.103– 0.2) | 0.013  0.01  (0.009–0.018) | 0.183  0.075  (0.059-0.307) |  |  |  | LC-MS/MS | (Y. Guo et al. 2013) |
| Kuwait | Al. Jahra, Al. Asma | Multiple (no identified industries) | 38 | 37±21 | $\bar{x}$  ME  (95%CI) | 2.42  1.41  (1.33 -3.51) | 11.17  7.33  (7.5–14.85) | 0.803  0.448  (0.535–1.07) |  |  |  | 0.185  0.163  (0.142–0.228) | 0.119  0.089  (0.089–0.149) | 0.182  0.137  (0.128-0.237) | 0.006  ND  (0.002 -0.011) | 0.32  0.22  (0.22-0.421) |  |  |  | LC-MS/MS | (Y. Guo et al. 2013) |
| Malysia | Kuala Lumpur | Multiple  (no identified industries) | 29 | 30±9 | $\bar{x}$  ME  (95%CI) | 0.778  0.263  (0.04-1.51) | 2.85  1.55  (1.0 – 4.7) | 0.171  0.112  (0.087–0.255) |  |  |  | 0.072  0.033  (0.026–0.118) | 0.082  0.036  (0.021 – 0.143) | 0.101  0.043  (0.037–0.168) | 0.012  0.006  (0.002-0.022) | 0.186  0.065  (0.075-0.297) |  |  |  | LC-MS/MS | (Y. Guo et al. 2013) |
| Poland | the Upper Silesia region | environmental tobacco smoke ETS, indoor coal burning | 30 | 8 | ME |  |  |  |  |  |  |  |  |  |  | ETS: from 0.602 to 1.400  non-ETS: from 0.388 to 0.799 |  |  |  | HPLC-FLD | (E. Siwińska et al. 1998) |
| Poland | NS | coke oven plant | 55 | between 22 and 55 | ME  (95%CI) | 46.2  (15.1-146.5) | 36.2  (12.6-111.6) | 27.6  (5.4-206.5) |  | 11.9  (3.2-41.7) | 6.0  (1.3-32.3) | 1.7  (0.4-5.3) | 3.7  (0.9-19.5) | 5.7  (1.1-32.3) | 0.7  (0.2-4.1) | 15.4  (3.7-82.0) | <1.4 |  | <1.0 | GC-MS | (Campo et al. 2010) |
| South Korea | national research | environmental tobacco smoke ETS, alcohol consumption and residence area (coastal, rural, urban) | 4702 | between 20 and ≥ 70 | GM  (95%CI) |  | 3.84  (3.57–4.11) |  |  |  |  |  |  |  |  | 0.15  (0.13–0.17) |  |  |  | HPLC-FLD | (Sul et al. 2012) |
| South Korea | national research | organic chemicals | 6418 | between 19 and ≥ 70 | GM  (95%CI) |  | 2.22  (2.10–2.34) | 0.27  (0.25–0.28) |  |  | 0.10  (0.09–0.10) |  |  |  |  | 0.15  (0.14–0.16) |  |  |  | LC-MS/MS | (Choi et al. 2017) |
| South Korea | Busan, Seoul, Yeosu | Multiple (no identified industries) | 60 | 35±11 | $\bar{x}$  ME  (95%CI) | 5.77  2.06  (2.87-8.66) | 9.98  5.87  (6.66-13.3) | 0.332  0.165  (0.216–0.448) |  |  |  | 0.093  0.066  (0.072–0.115) | 0.072  0.049  (0.053–0.091) | 0.118  0.078  (0.084–0.152) | 0.011  0.007  (0.008–0.014) | 0.167  0.103  (0.12-0.213) |  |  |  | LC-MS/MS | (Y. Guo et al. 2013) |
| South Korea | Seoul/Incheon, Pohang | environmental tobacco smoke ETS, heating fuel, environmental pollution | 654 | between 10 and 45 | $\bar{x}$ (SE)  ME (IQR) |  | 22.1 (1.16)  15.0 (8.72-25.56) |  |  |  |  |  |  |  |  |  |  |  |  | HPLC-FLD | (Yoon et al. 2012) |
| Sweden | southern Sweden | soot | 151 | between 19 and 66 | ME  (range) |  |  |  |  |  |  |  | 0.78  (0.09-14.56) |  |  | 0.56  (0.03-7.49) |  | 6.28  (0.46-71.70) | 4.75  (0.00-42.86 ) | LC-MS/MS | (Alhamdow et al. 2017) |
| United States | national research | NS | 3085 not obese;  1680 obese | between 45 and 47 | GM ± SE | not obese  2.823±0.012  obese  2.675±0.174 | not obese  3.270±0.139  obese  3.805±0.174 | not obese  0.322±0.013  obese  0.354±0.015 | not obese  0.136±0.006  obese  0.132±0.007 |  | not obese  0.144±0.004;  obese  0.167±0.005 |  | not obese  0.059±0.002  obese  0.075±0.003 | not obese  0.104±0.003  obese  0.106±0.004 |  | not obese  0.078±0.003  obese  0.085±0.004 |  |  |  | GC-MS/MS | (Ranjbar et al. 2015) |
| United States | Northern  California | environmental tobacco smoke ETS, diet, traffic density | 431 | between 6 and 8 | GM  (95%CI) | 1.51  (1.32–1.71) | 1.57  (1.42–1.74) | 0.14  (0.13–0.15) | 0.0618  (0.0571–0.0668) | 0.172  (0.16–0.186) | 0.0755  (0.0701–0.0814) |  | 0.0317  (0.0296–0.0341) | 0.0664  (0.0616–0.0716) | ND | 0.0742  (0.0689–0.08) |  |  |  | GC-MS | (Dobraca et al. 2018) |
| United States | Westbury, Atlanta | smoking | 72 | - | GM | smoker  6.81  non-smoker  0.57 | smoker  11.24  non-smoker  1.97 | smoker  0.76  non-smoker  0.09 | smoker  0.46  non-smoker  0.04 |  | smoker  1.13  non-smoker  0.05 |  | sum of 2-OH-PHEN and 3-OH-PHEN | | smoker  0.03  non-smoker  0.01 | smoker  0.38  non-smoker  0.08 |  |  |  | LC-MS/MS | (Wang et al. 2017) |
|  |  |  |  |  |  |  |  |  |  |  |  |  | smoker  0.33  non-smoker  0.08 | |  |  |  |  |  |  |  |
| United States | Boston | air inhalation | 370 | 36±5.5 | GM  (95%CI) | 2.82  (0.63-13.3) | 0.93  (<0.4-15.2) |  |  |  |  |  |  |  |  |  |  |  |  | GC-MS/MS | (Meeker et al. 2007) |
| Vietnam | Hanoi | Multiple (no identified industries) | 23 | 51± 19 | $\bar{x}$  ME  (95%CI) | 2.45  0.642  (0.177-0.417) | 15.31  4.9  (ND -34.39) | 0.639  0.473  (0.387–0.89) |  |  |  | 0.39  0.279  (0.212–0.569) | 0.237  0.157  (0.092–0.383) | 0.26  0.201  (0.161– 0.36) | 0.062  0.032  (ND –0.125) | 0.641  0.463  (0.31-0.973) |  |  |  | LC-MS/MS | (Y. Guo et al. 2013) |

Table S3a. Review of urinary concentrations of low molecular weight OH-PAHs (OH-naphthalene – OH-pyrene) expressed in µg/g creatinine, depending on the country of determination

| **country** | **location** | **source of exposure** | **study population** | **age**  **mean ±SD** | **statistic** | **OH-PAHs concentration** | | | | | | | | | | |  |  |
| --- | --- | --- | --- | --- | --- | --- | --- | --- | --- | --- | --- | --- | --- | --- | --- | --- | --- | --- |
|  |  |  |  |  |  | **1-OH-NAP** | **2-OH-NAP** | **2-OH-FL** | **3-OH-FL** | **9-OH-FL** | **1-OH-PHEN** | **9-OH-PHEN** | **2-OH-PHEN** | **3-OH-PHEN** | **4-OH-PHEN** | **1-OH-PYR** | **Analytical method** | **Reference** |
|  |  |  |  |  |  | **creatinine corrected (µg/g creatinine)** | | | | | | | | | | |  |  |
| Brasil | Jandaíra | exposed to the artisanal cashew nut roasting pollutants | 77 | 29.9 ± 11.9 | $\bar{x}$  (range) |  |  |  |  |  |  |  |  |  |  | 0.060  (0.025–0.421) | HPLC-FLD | (de Oliveira Galvão et al. 2017) |
| Canada | Shawinigan; Trois- Rivieres, | aluminium reduction plant and status smoker | 40 | between 30 and 45 | GM  (range) |  |  |  |  |  |  |  |  |  |  | from 0.089 to 0.482  (0.023-0.864) | HPLC-FLD | (Gilbert and Viau 1997) |
| Canada | Québec | wood treatment plant, silicon carbide plant | 140 | adult | GM  (95% CI) |  |  |  |  |  |  |  |  |  |  | 0.154  (0.039-0..617) | HPLC, GC-MS | (Viau et al. 1995) |
| Canada | Hamilton | air pollution (mostly steel fabrication) | 19 | 32.8±6.3 | GM±SD  (range) | 1.411±0.002  (0.160–8.232) | 2.605±0.002  (0.320–10.523) | 0.216±0.002  (0.088–3.768) | 0.048±0.002  (0.009–0.652) | 0.391±.0002  (0.145–2.557) | 0.244±0.002  (0.086–0.909) |  | 0.082±0.002  (0.036–0.356) | 0.070±0.002  (0.021–0.240) | 0.040±0.002  (0.013–0.133) | 0.136 ±0.002  (0.049–0.531) | GC-MS | (Nethery et al. 2012) |
| China | Nankin | air pollution | 768 | 29.32±3.14 | GM  (95%CI) | from 1.98 to 2.19  (1.76–2.45) | from 3.60 to 4.32  (3.19–4.92) | from 2.52 to 2.90  (2.27–3.21 |  |  |  |  |  |  |  | from 1.06 to 1.25 (0.95 1.43) | LC-MS/MS | (Xia et al. 2009) |
| China | Nanjing | NS | 480 | 32.01 ± 16.09 | GM  (95%CI) | 2.078  (1.907–2.265) | 4.064  (3.740–4.415) | 2.822  (2.630–3.028) |  |  |  |  |  |  |  | 1.156  (1.075–1.243) | LC-MS/MS | (Zhu et al. 2009) |
| China | northern  China | coke oven plant | 348 | between 21 and 55 | ME  (95%CI) |  | 910.026  (227.506-2970.223) | 467.148  (193.303-1707.507) |  |  |  | 154.532  (68.681-703.981) |  |  |  | 115.762  (38.587-733.159) | HPLC-FLD | (J. Yang et al. 2018) |
| China | not indicated | coke oven plant | 13 | adult | $\bar{x}$±SD  (range) | 25.107±12.503  (11.432-50.979) | 30.078±7.813  (17.970-40.911) | 38.016±20.619  (16.753-69.106) |  |  |  | 11.023±7.297  (4.808-23.352) |  | 22.836±814.165  (11.229-50.309) |  | 16.361±13.834  (4.245-37.430) | LC-MS/MS | (Xu Xu et al. 2004) |
| China | Wuhan | NS | 2824 | 51.6 ± 14.5 | ME | from 5.417 to 6.067 | from 11.139 to 12.222 | from 2.610 to 2.964 |  | from 8.634 to 10.374 | from 2.112 to 2.576 |  | from 0.002 to 0.003 | from 2.988 to 3.520 | from 6.233 to 7.315 | from 6.502 to 6.695 | GC-MS | (L. Yang et al. 2014) |
| Czech Republic | Hradec Králové | air pollution | 57 | between 6 and 8 | GM  (range) |  |  |  |  |  |  |  |  |  |  | from 0.108 to 0.205  (0.019-2.751) | HPLC-FLD | (Fiala et al. 2001) |
| Denmark | Copenhagen and villages situated in 20–50 km outside of Copenhagen | air pollution | 202 | between 3 and 13 | ME  $\bar{x}$  (range) |  |  |  |  |  |  |  |  |  |  | city:  0.154  0.211  (0.019–0.830)  country:  0.116  0.174  (0.019- 1.138) | HPLC-FLD | (Hansen et al. 2006) |
| Denmark | Amsterdam, Rotterdam, Laura, Eikske | air and soil pollution | 200 | between 1 and 6 | GM |  |  |  |  |  |  |  |  |  |  | from 0.347 to 0.482 | HPLC-FLD | (Van Wijnen et al. 1996) |
| Finland | Helsinki suburb | asphalt paving workers | 24 | 40 ± 21 | GM  (range) | from 16.568 to 21.666  (19.117-33.137) | from 1.274 to 12.108  (1.032-29.313) |  |  |  | sum of 1-OH-PHEN and 9-OH-PHEN | | from <0.275 to 1.116  (<0.275-2.060) | from <0.223 to 0.687  (<0.223-0.859) |  | from <0.116 to 1.312  (<0.116-7.525) | HPLC-FLD | (Väänänen et al. 2003) |
|  |  |  |  |  |  |  |  |  |  |  | from <0.309 to 2.404  (<0.309-3.949) | |  |  |  |  |  |  |
| France | Grenoble and suburbs | smoking | 36 | between 18 and 65 | $\bar{x}$ ±SD  ME  (range) |  |  |  |  |  |  |  |  |  |  | smoker  0.313±0.174  0.257  (0.064-0.637)  non-smoker  0.081±0.064  0.073  (0.014-0.322) | HPLC-FLD | (Barbeau et al. 2011) |
| France | the Nord-Pas de Calais region of northern France | metallurgical industries, urban setting | 25 | 29 | ME  (range) |  |  |  |  |  |  |  |  |  |  | from 0.075 to 0.080  (0.009-0.416) | HPLC-FLD | (Leroyer et al. 2010) |
| France | Paris or its suburbs | smoking | 54 | between 18 and 65 | $\bar{x}$  ME  (range) |  |  |  |  |  |  |  |  |  |  | smoker  0.278  0.291  (0.079-1.023)  non-smoker  0.085  0.062  (0.19-0.286) | HPLC-FLD | (Lafontaine et al. 2006) |
| Germany | Frankfurt am Main | the parquet glue | 382 | between 0 and ≥20 | $\bar{x}$±SD  ME |  |  |  |  |  | 0.436±0.272  0.372 |  | 0.252±0.188  0.198 | 0.374±0.245  0.320 | 0.061±0.151  0.034 | 0.143±0.131  0.115 | HPLC-FLD | (Heudorf and Angerer 2001a) |
| Germany | Frankfurt am Main | smoking | 495 | 37.9±7.9 | $\bar{x}$±SD  ME |  |  |  |  |  | smoker  0.437±0.311  0.357  non-smoker  0.445±0.358  0.350 |  | smoker  0.314±0.216  0.240  non-smoker  0.266±0.231  0.206 | smoker  0.473±  0.3020.389  non-smoker  0.305±0.209  0.244 | smoker  0.065±0.140  0.039  non-smoker  0.058±0.170  0.030 | smoker  0.195±0.142  0.152  non-smoker  0.100±0.101  0.077 | HPLC-FLD | (Heudorf and Angerer 2001b) |
| Germany | southern Germany | occupational exposure: Carbon electrode production, Road stone impregnation, Aluminium smelting, Glass manufacturing,  Chimney-sweeping, Meat smoking, Municipal and Industrial waste incineration | 232 | adult | ME  (range) |  |  |  |  |  |  |  |  |  |  | from 0.11 to 24.5  (<0.1-126.2) | HPLC-FLD | (Göen T., Gündel J., Schaller K.-H. 1995a) |
| Germany | southern Germany | urban area | 107 | between 2 and 64 a | ME  $\bar{x}$  (range) | 4.5  8.3  (<LOD-67.1) | 4.4  6.7  (<LOD-48.3) |  |  |  |  |  |  |  |  |  | HPLC-FLD | (Preuss et al. 2004) |
| Iran | Shiraz | environmental tobacco smoke ETS, dietary | 115 | 10.71 ± 1.15 | GM  ME  (range) |  |  |  |  |  |  |  |  |  |  | 1.460  (0.120-6.950) | HPLC-FLD | (Shahsavani et al. 2017) |
| Japan | suburb of metropolitan Tokyo | environmental tobacco smoke ETS | 134 | 4.7±0.9 | GM ±SD  ME |  |  |  |  |  |  |  |  |  |  | 0.127±1.83  0.127 | HPLC-FLD | (Mori et al. 2011) |
| Japan | Kanazawa | smoking | 17 | between 22 and 69 | $\bar{x}$±SD  (range) |  |  | smoker:  0.370±0.226  (0.097–0.757)  non-smoker:  0.064±0.032  (0.016–0.097) |  |  |  |  |  |  |  | smoker:  0.232±0.251 (0.039–0.868)  non-smoker:  0.116 ± 0.154 (0.019–0.424) | HPLC-FLD | (Chetiyanukornkul et al. 2004) |
| Netherlands | national research | petrochemical industry | 562 | adult | ME  $\bar{x}$  (range) |  |  |  |  |  |  |  |  |  |  | from 0.328 to 2.817  from 0.193 to 1.505  (<0.096–45.340) | HPLC-FLD | (Boogaard and van Sittert 1995) |
| Netherlands | NS | coke oven plant | 35 | 38±6 | $\bar{x}$±SD  ME |  |  |  |  |  |  |  |  |  |  | 2.392±2.045  1.794 | HPLC-FLD | (Van Delft et al. 2001) |
| New Zealand | Christchurch | air pollution | 89 | between 12 and 18 | ME  (range) |  |  |  |  |  |  |  |  |  |  | from 0.037 to 0.083  (0.008–1.397) | HPLC-FLD | (Cavanagh et al. 2007) |
| Mexico | San Luis Potosi | environmental pollution | 206 | 47.2±21.0 | GM±SD |  |  |  |  |  |  |  |  |  |  | 2.643±4.013 | HPLC-FLD | (Ochoa-Martínez et al. 2017) |
| Mexico | San Vicente, Tercera, Ramonal, Ventanilla, Victoria, Tancuime, San Luis Potosi, El Centro and Domingo | air pollution (industrial activity, indoor wood combustion, waste disposal and brick manufacturing) | 258 | between 3 and 13 | $\bar{x}$±SD |  |  |  |  |  |  |  |  |  |  | from 0.154±0.039 to 8.894±7.139 | HPLC-FLD | (Martínez-Salinas et al. 2010) |
| Mexico | Ciudad Juarez | environmental chemicals | 100 | 7±2.5 | $\bar{x}$±SD  (range) |  |  |  |  |  |  |  |  |  |  | 2.315±2.122  (<0.019-7.525) | HPLC-FLD | (Ochoa-Martinez et al. 2016) |
| Poland | Krakow | outdoor and indoor air pollution | 218 | 3 | GM  (95%CI) | 3.838  (3.413–4.315) | 8.449  (7.652–9.33) | 0.942  (0.879–1.01) | 0.366  (0.335–0.4) | 0.998  (0.923–1.079) | 0.639  (0.597–0.683) |  | 0.169  (0.159–0.181) | 0.403 (0.374–0.434) | 0.104 (0.096–0.112) | 0.357 (0.331–0.385) | GC-MS | (Sochacka-Tatara et al. 2018) |
| Poland | the Upper Silesia region | Coal heating and/or cooking;  environmental tobacco smoke ETS;  environmental pollutants | 412 | between 7 and 8 | $\bar{x}$±SD |  |  |  |  |  |  |  |  |  |  | from 0.679±0.133 to 1.136±0.135 | HPLC-FLD | (Ewa Siwińska et al. 1999) |
| Poland | the Upper Silesia region | emissions from coal-burning stoves and environmental tobacco smoke ETS | 66 | between 5 and 14 | $\bar{x}$±SD  ME  (range) |  |  |  |  |  |  |  |  |  |  | 1.042±0.598  0.888  (0.077–3.762) | HPLC-FLD | (Mielzyńska et al. 2006) |
| Portugal | Paranhos, Chaves | inhalation and dermal PAH exposures | 43 | between 3 and 5 | ME  (range) | 1-OH-NAP + 1-OH-ACE | from 1.035 to 0.446  (0.001-7.367) |  |  |  | from 0.055 to 0.294  (0.005-1.004) |  |  |  |  | from 0.043 to 0.345  (0.005-1.816) | HPLC-FLD | (Oliveira et al. 2016) |
|  |  |  |  |  |  | from 0.664 to 9.35  (0.0511-17.2)  (μmol/mol creatinine) |  |  |  |  |  |  |  |  |  |  |  |  |
| Saudi Arabian | Jeddah | refinery, highway, Red sea | 204 | between 10 and 12 | ME |  |  |  |  |  | from 0.169 to 0.209 | sum of 2-OH-PHEN and 9-OH-PHEN | | from 0.146 to 0.217 | from 0.035 to 0.048 | from 0.189 to 0.339 | HPLC-FLD | (Alghamdi et al. 2015) |
|  |  |  |  |  |  |  |  |  |  |  |  | from 0.080 to 0.100 | |  |  |  |  |  |
| South Korea | not indicated | work in aircraft maintenance, smoking and consumption of grilled food |  | 32.17±10.3 | GM±SD |  | 4.142±2.447 |  |  |  |  |  |  |  |  | 0.617 ±3.550 | HPLC-FLD | (Lee et al. 2001) |
| South Korea | Yuchun, Seou | air pollution (heavy traffic) | 137 | between 11 and 13 | GM  ME |  | 3.148  2.791 |  |  |  |  |  |  |  |  | 0.193  0.174 | HPLC-FLD | (Kang et al. 2002) |
| Spain | Granada province | air pollution | 174 | 4.3 ± 0.2 | $\bar{x}$  (range) |  |  |  |  |  |  |  |  |  |  | exposed to ETS:  0.122  non- exposed to ETS:  0.100 | HPLC-FLD | (Freire et al. 2009) |
| Spain | Valencia and surroundings | air pollution including smoking status | 204 | between 16 and ≥ 36 | ME |  |  |  |  |  |  |  |  |  |  | smoker: 0.183  non-smokre: 0.096 | HPLC-FLD | (Llop et al. 2008) |
| Taiwan | Kaoshiung city;  Nanto village | petrochemical industry | 72 | 11 | $\bar{x}$  (range) |  |  |  |  |  |  |  |  |  |  | city  0.156  (0.015-0.449)  village  0.110  (0.017–0.241) | HPLC-FLD | (Kuo et al. 2004) |
| Taiwan | not indicated | Environmental tobacco smoke (ETS) | 40 | between 3 and 6 | $\bar{x}$±SD  (range) |  |  |  |  |  |  |  |  |  |  | morning: 0.069±0.192 (0.002–1.019)  evening: 0.073±0.221  (0.002–1.328) | HPLC-FLD | (Tsai et al. 2003) |
| Thailand | Chiang Mai | smoking | 13 | between 26 and 76 | $\bar{x}$±SD  (range) |  |  | smoker:  1.208±0.242  (0.805-1.627)  non-smoker:  0.354± 0.242 (0.081–0.741) |  |  |  |  |  |  |  | smoker:  5.846±3.685 (1.929–11.113  non-smoker:  1.756±1.138 (0.386–3.319) | HPLC-FLD | (Chetiyanukornkul et al. 2004) |
| Thailand | Chiang Mai Province | air pollution (smoke haze episode) | 200 | 11.7 ± 1.9 | ME  (range) |  |  |  |  |  |  |  |  |  |  | 0.984  (0.810-16.361) | HPLC-FLD | (Naksen et al. 2017) |
| Thailand | Chiang Mai | farmers, taxi drivers and traffic policeman | 30 | between25 and 62 | $\bar{x}$±SD  (range) | from 5.952±2.167 to 9.661±6.054  (1.924-18.964) | from 2.906±2.23 to 15.421±7.647  (0.650-25.706) | from 0.548±0.322 to 4.220±2.336  (0.161-8.167) |  |  | sum of 1-OH-PHEN and 9-OH-PHEN | | from 0.189±0.137 to 1.408±0.704  (0.069-2.198) | from 0.189±0.120 to 1.975±0.584  (0.007-2.679) | from 0.034±0.017 to 0.155±0.103  (0.017-0.326) | from 0.347±0.251 to 2.315±1.351  (0.116-4.534) | HPLC-FLD | (Chetiyanukornkul et al. 2006) |
|  |  |  |  |  |  |  |  |  |  |  | from 0.172±0.120 to 0.876±0.876  (0.034-1.511) | |  |  |  |  |  |  |
| Turkey | Ankara | work in engine repair workshops | 61 | between 13 and 18 | $\bar{x}$±SD  (range) |  |  |  |  |  |  |  |  |  |  | 9.087±1.023  (1.543–46.112) | HPLC-FLD | (Karahalil et al. 1998) |
| Ukraine | Kiev;  Mariupol | high traffic urban areas;  two steel plants and an associated coking facility | 92 | 3 | $\bar{x}$±SD  median |  |  |  |  |  |  |  |  |  |  | Kiev: 0.656±0.386  0.540  Mariupol:  1.331±0.965  0.945 | HPLC-FLD | (Mucha et al. 2006) |
| United States | national research | non-occupational exposure | 2400 | between 9 and ≥20 | GM  (95%CI) |  |  | 0.408  (0.302-0.551) | 0.157  (0.118–0.210) |  | 0.420  (0.116-0.173) |  | 0.090  (0.076–0.107) | 0.116  (0.102–0.133) |  | 0.074  0.064–0.086) | GC-MS | (Grainger et al. 2006) |
| United States | national research | Multiple | 2748 | between 9 and ≥20 | GM (95%CI) | 1.910  (1.700-2.160) | 2.310  (1.980-2.300) | 0.298  (0.361-0.340) | 0.125  (0.108-0.144) | 0.205  (0.183-0.229) | 0.132  (0.118-0.147) | 0.032  (0.028-0.036) | 0.051.6  (0.048-0.067) | 0.098  (0.087-0.110) | 0.039  (0.030-0.052) | 0.046  (0.041-0.053) | GC-MS | (Li et al. 2008) |
| United States | national research | NS | 3283 | between 20 and ≥ 60 | $\bar{x}$ | 0.08 | 0.008 | 0.006 | 0.005 |  | 0.005 |  | 0.004 | 0.005 |  | 0.004 | GC-MS | (Xiaohui Xu et al. 2010) |
| United States | national research | NS | 1768 | 47.65 ± 16.71 | GM  (95%CI) | 2.984  (2.612-3.407) | 4.294  (3.980-4.633) | 0.309  (0.283-0.337) | 0.119  (0.108-0.131) | 0.326  (0.307-0.346) | 0.160  (0.153-0.168) |  | 0.070  (0.066-0.074) | 0.092  (0.087-0.097) |  | 0.114  (0.107-0.121) | GC-MS/MS | (J. Guo et al. 2018) |
| United States | Atlanta | grilled or smoked food | 9 | 37 ± 12 | ME  (range) | 70.1  (40.1-94.3) | 61.2  (49.8-116) | 5.57  (3.85-7.26) | 2.24  (1.69-3.1) | 12.2  (4.28-18.4) | 2.94  (1.71-4.02) |  | 2.19  (1.63-3.21) | 3.04  (2.06-3.45) | 0.71  (0.45-2.02) | 1.86  (1.57-2.95) | GC-MS | (Li et al. 2012) |
| United States | Atlanta | inhaled indoor air and consumed food | 8 | between 31 and 62 | ME  (95%CI) | 1.469  (0.349–11.896) | 2.109  (0.715–6.227) | 0.173  (0.061–0.466) | 0.066  (0.023–0.268) | 0.247  (0.071–1.532) | 0.125  (0.046–0.348) |  | 0.051  (0.023–0.164) | 0.076  (0.034-0.281) |  | 0.067  (0.024–0.261) | GC-MS | (Li et al. 2010) |
| United States | downtown Durham, North Carolina | inhalation, nondietary ingestion, dietary ingestion | 48 | between 2 and 4 | $\bar{x}$±SD  (range) |  |  |  |  |  |  |  |  |  |  | adult:  0.093±0.106  (0.004-0.521)  child:  0.096±0.079  (0.015-0.347) | GC-MS | (Chuang et al. 1999) |
| United States | US Air Force (USAF) | inhalation of jet propulsion fuel | 73 | between 18 and 43 | GM±SD | from 0.813±0.002 to  6.006±0.002 | from 2.034±0.002  to 6.376±0.002 | from 0.163±0.002 0.696±0.002 | from 0.059±0.002 to 0.337±0.002 | from 0.198±0.002 to 0.373±0.002 | from 0.099±0.002 to 0.149±0.002 |  | from 0.042±0.002 to 0.079±0.002 | from 0.057±0.002 to 0.147±0.002 | from 0.017±0.002 to 0.029±0.002 | from 0.0±0.002 to 0.122±0.002 | GC-MS | (Rodrigues et al. 2014) |
| United States | Greater Boston area | inhalation and dermal PAH exposures among highway builders | 26 | adult | $ME$ |  |  |  |  |  |  |  |  |  |  | from 0.12 to 1.2 | HPLC-FLD | (McClean et al. 2004) |
| United States | NS | woodsmoke | 9 | between 20 and 65 | ME  (range) | 7.32  (4.47–12.6) | 6.3  (2.88–9.73 | 0.51  (0.26–0.62) | 0.16  (0.09–0.22) | 0.9  (0.56–2.19) | 0.22  (0.15–0.33) |  | 0.14  (0.07–0.24) | 0.17  (0.1–0.25) |  | 0.16  (0.06–0.3) | GC-MS | (Li et al. 2016) |
| United States | Ohio | lifestyle factors (i.e. time spent outdoors, household smoking habits, cooking appliances, and cooking habits) | 126 | between 2 and 5 | GM  (range) |  |  |  |  |  |  |  |  |  |  | 0.44  (<0.2-4.1) | GC-MS | (Morgan et al. 2015) |

Table S3b. Review of urinary concentrations of occasionally determined OH-PAHs expressed in µg/g creatinine depending on the country

| **country** | **location** | **source of exposure** | **study population** | **age**  **mean ±SD** | **statistic** | **OH-PAHs concentration** | | | | | | | | |  |  |
| --- | --- | --- | --- | --- | --- | --- | --- | --- | --- | --- | --- | --- | --- | --- | --- | --- |
|  |  |  |  |  |  | **3-OH- FRT** | **3-OH-CHRY** | **6-OH-CHRY** | **1-OH-BaA** | **3-OH-BaA** | **1-OH-BaP** | **3-OH-BaP** | **9-OH-BaP** | **6-OH-IndPy** | **Analytical method** | **Reference** |
|  |  |  |  |  |  | **creatinine corrected (µg/g creatinine)** | | | | | | | | |  |  |
| China | not indicated | coke oven plant | 13 | adult | $\bar{x}$±SD  (range) | 0.014±0.017  (0.004-0.052) | 1.382 ±1.132  (0.432-3.455) | 0.022±0.013  (0.002-0.032) |  |  |  | 0.085±0.168  (0.002-0.465) | 0.451 |  | LC-MS/MS | (Xu Xu et al. 2004) |
| France | Grenoble and suburbs | smoking | 36 | between 18 and 65 | $\bar{x}$ ±SD  ME  (range) |  |  |  |  |  |  | smoker  0.0001±0.00006  0.0002  (0.00007-0.0002)  non-smoker  0.0002 ±0.00001  0.0001  (0.00001-0.0001) |  |  | HPLC-FLD | (Barbeau et al. 2011) |
| France | the Nord-Pas de Calais region of northern France | metallurgical industries, urban setting | 25 | 29 | ME  (range) |  |  |  |  |  |  | (<0.00021-0.00023) |  |  | HPLC-FLD | (Leroyer et al. 2010) |
| France | Paris or its suburbs | smoking | 54 | between 18 and 65 | $\bar{x}$  ME  (range) |  |  |  |  |  |  | smoker  0.071  0.055  (<0.024-0.199)  non-smoker  0.033  0.026  (<0.024-0.107) |  |  | HPLC-FLD | (Lafontaine et al. 2006) |
| United States | downtown Durham, North Carolina | inhalation, nondietary ingestion, dietary ingestion | 48 | between 2 and 4 | $\bar{x}$±SD  (range) | adult:  0.135±0.193  (0.017-0.810)  child:  0.0123±0.137  (0.077-0.579) |  | adult:  0.160±0.216  (0.009-1.296)  child:  0.050±0.041  (0.009-0.158) | adult:  0.022±0.324  (0.015-1.555)  child:  0.099±0.089  (0.011-0.367) | adult:  0.346±0.410  (0.011-1.771)  child:  0.060±0.041  (0.009-0.158) | sum of 1-OH-BaP and 3-OH-BaP | |  | adult:  0.014±0.362  (0.003-1.731)  child:  0.034±0.026  (0.003-0.111) | GC-MS | (Chuang et al. 1999) |
|  |  |  |  |  |  |  |  |  |  |  | adult:  0.052±0.109  (0.005-0.403)  child:  0.066±0.007  (0.012-0.379) | |  |  |  |  |

Table S4a. Summary of parameters of analytical methods used to determine urinary OH-PAHs by liquid chromatography in works reported in the review

|  | **HPLC-FLD** | | | | | | **LC-MS/MS** | | | | | | | |
| --- | --- | --- | --- | --- | --- | --- | --- | --- | --- | --- | --- | --- | --- | --- |
|  | **LOD**  **(μg/l)** | **References** | **LOQ**  **(μg/l)** | **References** | **Recovery (%)** | | **LOD**  **(μg/l)** | **References** | **Recovery (%)** | | | | | |
|  |  |  |  |  | **C18 SPE cartridge** | **References** |  |  | **Sep-Pak C18**  **SPE cartridge** | **References** | **Oasis WAX on-line**  **SPE**  **cartridge** | **References** | **SUPELCO**  **SPE**  **cartridges** | **References** |
| **1-OH-NAP** | 1.5 | (Preuss et al. 2004) |  |  |  |  | 0.06 – 0.5 | (Wang et al. 2017; Xia et al. 2009; Zhu et al. 2009) | 70 - 80 | (Xu Xu et al. 2004) | 67 | (Wang et al. 2017) | 81 - 123 | (Zhu et al. 2009) |
| **2-OH-NAP** | 0.03 – 0.5 | (Kang et al. 2002; Preuss et al. 2004; Sul et al. 2012; J. Yang et al. 2018; Yoon et al. 2012) |  |  |  |  | 0.033 – 0.5 | (Liu et al. 2017; Wang et al. 2017; Xia et al. 2009; Zhu et al. 2009) | 70 - 80 | (Xu Xu et al. 2004) | 67 | (Wang et al. 2017) | 81 - 123 | (Zhu et al. 2009) |
| **2-OH-FL** | 0.04 – 0.12 | (J. Yang et al. 2018) |  |  |  |  | 0.008 - 1 | (Liu et al. 2017; Wang et al. 2017; Xia et al. 2009; Zhu et al. 2009) | 70 - 80 | (Xu Xu et al. 2004) | 64 | (Wang et al. 2017) | 81 - 123 | (Zhu et al. 2009) |
| **3-OH-FL** |  |  |  |  |  |  | 0.008 | (Wang et al. 2017) | 70 - 80 | (Xu Xu et al. 2004) | 81 | (Wang et al. 2017) |  |  |
| **9-OH-FL** |  |  |  |  |  |  |  |  | 70 - 80 | (Xu Xu et al. 2004) |  |  |  |  |
| **1-OH-PHEN** | 0.012 - 0.016 | (Becker et al. 2002; Hemat et al. 2012; Heudorf and Angerer 2001a, 2001b; Schulz et al. 2008) |  |  |  |  | 0.01 | (Wang et al. 2017) |  |  | 72 | (Wang et al. 2017) |  |  |
| **2-OH-PHEN** | 0.004 – 0.012 | (Becker et al. 2002; Hemat et al. 2012; Heudorf and Angerer 2001a, 2001b; Schulz et al. 2008) |  |  |  |  | 0.01 - 0.05 | (Alhamdow et al. 2017; Wang et al. 2017) |  |  | 63 | (Wang et al. 2017) |  |  |
| **3-OH-PHEN** | 0.005 – 0.012 | (Becker et al. 2002; Hemat et al. 2012; Heudorf and Angerer 2001a, 2001b; Schulz et al. 2008) |  |  |  |  |  |  |  |  |  |  |  |  |
| **4-OH-PHEN** | 0.005 - 0.008 | (Hemat et al. 2012; Heudorf and Angerer 2001a, 2001b; Schulz et al. 2008) |  |  |  |  | 0.007 | (Wang et al. 2017) |  |  | 55 | (Wang et al. 2017) |  |  |
| **9-OH-PHEN** | 0.004 – 0.12 | (Becker et al. 2002; Hemat et al. 2012; Schulz et al. 2008; J. Yang et al. 2018) |  |  |  |  | 0.018 | (Liu et al. 2017) |  |  |  |  |  |  |
| **1-OH-PYR** | 0.005 – 0.72 | (Barbeau et al. 2011; Becker et al. 2002; Cavanagh et al. 2007; de Oliveira Galvão et al. 2017; Göen T., Gündel J., Schaller K.-H. 1995b; Hemat et al. 2012; Heudorf and Angerer 2001a, 2001b; Kang et al. 2002; Kuo et al. 2004; Lafontaine et al. 2006; Leroyer et al. 2010; Llop et al. 2008; Martínez-Salinas et al. 2010; McClean et al. 2004; Mielzyńska et al. 2006; Naksen et al. 2017; Ochoa-Martinez et al. 2016; Schulz et al. 2008; Shahsavani et al. 2017; E. Siwińska et al. 1998; Sul et al. 2012; Van Wijnen et al. 1996; J. Yang et al. 2018) | 0.03 - 0.079 | (Freire et al. 2009; Martínez-Salinas et al. 2010) | 88 - 102 | (Cavanagh et al. 2007; Martínez-Salinas et al. 2010; McClean et al. 2004; Ochoa-Martinez et al. 2016; Ochoa-Martínez et al. 2017; E. Siwińska et al. 1998) | 0.006 – 0.5 | (Alhamdow et al. 2017; Liu et al. 2017; Wang et al. 2017; Xia et al. 2009; Zhu et al. 2009) | 70 - 80 | (Xu Xu et al. 2004) | 49 | (Wang et al. 2017) | 81 - 123 | (Zhu et al. 2009) |
| **3-OH- FRT** |  |  |  |  |  |  |  |  | 70 - 80 | (Xu Xu et al. 2004) |  |  |  |  |
| **3-OH-CHRY** |  |  |  |  |  |  |  |  | 70 - 80 | (Xu Xu et al. 2004) |  |  |  |  |
| **6-OH-CHRY** |  |  |  |  |  |  |  |  | 70 - 80 | (Xu Xu et al. 2004) |  |  |  |  |
| **3-OH-BcPHEN** |  |  |  |  |  |  |  |  | 70 - 80 | (Xu Xu et al. 2004) |  |  |  |  |
| **1-OH-BaA** |  |  |  |  |  |  |  |  | 70 - 80 | (Xu Xu et al. 2004) |  |  |  |  |
| **3-OH-BaA** |  |  |  |  |  |  | 0.002 | (Alhamdow et al. 2017) |  |  |  |  |  |  |
| **3-OH-BaPYR** | 0.000008 – 0.00005 | (Barbeau et al. 2011; Lafontaine et al. 2006; Leroyer et al. 2010) | 0.00005 – 0.00025 | (Barbeau et al. 2011; Leroyer et al. 2010) |  |  | 0.002 | (Alhamdow et al. 2017) | 70 - 80 | (Xu Xu et al. 2004) |  |  |  |  |
| **9-OH-BaPYR** |  |  |  |  |  |  |  |  | 70 - 80 | (Xu Xu et al. 2004) |  |  |  |  |

Table S4b. Summary of parameters of analytical methods used to determine urinary OH-PAHs by gas chromatography in works reported in the review

|  | **GC - MS** | | | | | | | | **GC-MS/MS** | |
| --- | --- | --- | --- | --- | --- | --- | --- | --- | --- | --- |
|  | **LOD**  **(μg/l)** | **References** | **LOQ**  **(μg/l)** | **References** | **Recovery (%)** | | | | **LOD**  **(μg/l)** | **References** |
|  |  |  |  |  | **C18 SPE cartridges** | **References** | **EnvirElut PAH**  **SPE cartridges** | **References** |  |  |
| **1-OH-NAP** | 0.0026 - 0.9 | (Dobraca et al. 2018; Li et al. 2006, 2008, 2012, 2016; Rodrigues et al. 2014; Sochacka-Tatara et al. 2018; Xiaohui Xu et al. 2010; L. Yang et al. 2014) | 0.9 | (Campo et al. 2008, 2010) | 53 | (Campo et al. 2008) |  |  | 0.04 – 0.4 | (J. Guo et al. 2018; Y. Guo et al. 2013; Khoury et al. 2018; Meeker et al. 2007; Thai et al. 2016) |
| **2-OH-NAP** | 0.0024 - 0.9 | (Dobraca et al. 2018; Li et al. 2006, 2008, 2012, 2016; Rodrigues et al. 2014; Sochacka-Tatara et al. 2018; Xiaohui Xu et al. 2010; L. Yang et al. 2014) | 0.9 - 1.0 | (Campo et al. 2008, 2010) | 72 | (Campo et al. 2008) |  |  | 0.04 – 0.4 | (J. Guo et al. 2018; Y. Guo et al. 2013; Khoury et al. 2018; Meeker et al. 2007; Thai et al. 2016) |
| **2-OH-FL** | 0.0026 – 0.9 | (Dobraca et al. 2018; Grainger et al. 2006; Li et al. 2006, 2008, 2012, 2016; Rodrigues et al. 2014; Sochacka-Tatara et al. 2018; Xiaohui Xu et al. 2010; L. Yang et al. 2014) | 0.1 - 0.2 | (Campo et al. 2008, 2010) | 94 | (Campo et al. 2008) | 40 - 70 | (Grainger et al. 2006) | 0.003 - 0.01 | (J. Guo et al. 2018; Y. Guo et al. 2013; Khoury et al. 2018; Thai et al. 2016) |
| **3-OH-FL** | 0.002 – 0.075 | (Dobraca et al. 2018; Grainger et al. 2006; Li et al. 2006, 2008, 2012, 2016; Rodrigues et al. 2014; Sochacka-Tatara et al. 2018; Xiaohui Xu et al. 2010) |  |  |  |  | 40 -70 | (Grainger et al. 2006) | 0.001 - 0.01 | (J. Guo et al. 2018; Khoury et al. 2018; Thai et al. 2016) |
| **9-OH-FL** | 0.0026 - 0.9 | (Dobraca et al. 2018; Li et al. 2006, 2008, 2012, 2016; Rodrigues et al. 2014; Sochacka-Tatara et al. 2018; L. Yang et al. 2014) | 0.2 | (Campo et al. 2008, 2010) | 85 | (Campo et al. 2008) |  |  | 0.003 - 0.01 | (J. Guo et al. 2018; Khoury et al. 2018; Thai et al. 2016) |
| **1-OH-PHEN** | 0.0026 – 0.9 | (Dobraca et al. 2018; Grainger et al. 2006; Li et al. 2006, 2008, 2012, 2016; Rodrigues et al. 2014; Sochacka-Tatara et al. 2018; Xiaohui Xu et al. 2010; L. Yang et al. 2014) | 0.3 | (Campo et al. 2008, 2010) | 78 | (Campo et al. 2008) | 40 - 70 | (Grainger et al. 2006) | 0.005 - 0.01 | (J. Guo et al. 2018; Khoury et al. 2018; Thai et al. 2016) |
| **2-OH-PHEN** | 0.0026 – 0.9 | (Dobraca et al. 2018; Grainger et al. 2006; Li et al. 2006, 2008, 2012, 2016; Rodrigues et al. 2014; Sochacka-Tatara et al. 2018; Xiaohui Xu et al. 2010; L. Yang et al. 2014) | 0.1 | (Campo et al. 2008, 2010) | 109 | (Campo et al. 2008) | 40 - 70 | (Grainger et al. 2006) | 0.003 - 0.01 | (J. Guo et al. 2018; Y. Guo et al. 2013; Khoury et al. 2018; Thai et al. 2016) |
| **3-OH-PHEN** | 0.0026 – 0.9 | (Dobraca et al. 2018; Grainger et al. 2006; Li et al. 2006, 2008, 2012, 2016; Rodrigues et al. 2014; Sochacka-Tatara et al. 2018; Xiaohui Xu et al. 2010; L. Yang et al. 2014) | 0.2 | (Campo et al. 2008, 2010) | 127 | (Campo et al. 2008) | 40 - 70 | (Grainger et al. 2006) | 0.003 - 0.01 | (J. Guo et al. 2018; Y. Guo et al. 2013; Khoury et al. 2018; Thai et al. 2016) |
| **4-OH-PHEN** | 0.0026 – 0.9 | (Dobraca et al. 2018; Li et al. 2006, 2008, 2012; Rodrigues et al. 2014; Sochacka-Tatara et al. 2018; L. Yang et al. 2014) | 0.1 | (Campo et al. 2008, 2010) | 83 | (Campo et al. 2008) |  |  | 0.001 - 0.01 | (J. Guo et al. 2018; Y. Guo et al. 2013; Khoury et al. 2018; Thai et al. 2016) |
| **9-OH-PHEN** | 0.0026 – 0.9 | (Li et al. 2006, 2008; L. Yang et al. 2014) | 0.2 | (Campo et al. 2008, 2010) | 50 | (Campo et al. 2008) |  |  | 0.004 - 0.01 | (Y. Guo et al. 2013; Khoury et al. 2018) |
| **1-OH-PYR** | 0.002 – 0.9 | (Chuang et al. 1999; Dobraca et al. 2018; Grainger et al. 2006; Li et al. 2006, 2008, 2012, 2016; Rodrigues et al. 2014; Sochacka-Tatara et al. 2018; Xiaohui Xu et al. 2010; L. Yang et al. 2014) | 0.5 | (Campo et al. 2008, 2010) | 89 | (Campo et al. 2008) | 40 - 70 | (Grainger et al. 2006) | 0.002 - 0.01 | (J. Guo et al. 2018; Y. Guo et al. 2013; Khoury et al. 2018; Thai et al. 2016) |
| **3-OH- FRT** | 0.017 - 0.0035 | (Chuang et al. 1999; Grainger et al. 2006) |  |  |  |  | 40 -70 | (Grainger et al. 2006) | 0.008 | (Khoury et al. 2018) |
| **1-OH-CHRY** | 0.0026 – 0.075 | (Li et al. 2006, 2008; Sochacka-Tatara et al. 2018) |  |  |  |  |  |  | 0.01 | (Y. Guo et al. 2013) |
| **2-OH-CHRY** | 0.0026 – 0.075 | (Li et al. 2006, 2008; Sochacka-Tatara et al. 2018) |  |  |  |  |  |  | 0.004 | (Khoury et al. 2018) |
| **3-OH-CHRY** | 0.0026 – 0.075 | (Grainger et al. 2006; Li et al. 2006, 2008; Sochacka-Tatara et al. 2018) |  |  |  |  | 40 - 70 | (Grainger et al. 2006) | 0.003 | (Khoury et al. 2018) |
| **4-OH-CHRY** | 0.0026 – 0.075 | (Li et al. 2006, 2008; Sochacka-Tatara et al. 2018) |  |  |  |  |  |  | 0.003 | (Khoury et al. 2018) |
| **5-OH-CHRY** | 0.01 – 0.075 | (Sochacka-Tatara et al. 2018) |  |  |  |  |  |  |  |  |
| **6-OH-CHRY** | 0.0026 – 0.017 | (Chuang et al. 1999; Grainger et al. 2006; Li et al. 2006, 2008) | 1.4 | (Campo et al. 2008, 2010) | 23 | (Campo et al. 2008) | 40 - 70 | (Grainger et al. 2006) | 0.006 – 0.01 | (Y. Guo et al. 2013; Khoury et al. 2018) |
| **1-OH-BcPHEN** | 0.0028 – 0.075 | (Grainger et al. 2006; Li et al. 2006, 2008; Sochacka-Tatara et al. 2018) |  |  |  |  | 40 - 70 | (Grainger et al. 2006) |  |  |
| **2-OH-BcPHEN** | 0.003 – 0.075 | (Grainger et al. 2006; Li et al. 2006, 2008; Sochacka-Tatara et al. 2018) |  |  |  |  | 40 - 70 | (Grainger et al. 2006) |  |  |
| **3-OH-BcPHEN** | 0.0026 0.0054 | (Grainger et al. 2006; Li et al. 2006, 2008) |  |  |  |  | 40 - 70 | (Grainger et al. 2006) | 0.01 | (Y. Guo et al. 2013) |
| **1-OH-BaA** | 0.0026 – 0.075 | (Chuang et al. 1999; Grainger et al. 2006; Li et al. 2006, 2008; Sochacka-Tatara et al. 2018) |  |  |  |  | 40 - 70 | (Grainger et al. 2006) | 0.01 | (Y. Guo et al. 2013) |
| **3-OH-BaA** | 0.0052 – 0.075 | (Chuang et al. 1999; Grainger et al. 2006; Li et al. 2006, 2008; Sochacka-Tatara et al. 2018) |  |  |  |  | 40 - 70 | (Grainger et al. 2006) |  |  |
| **1-OH-BaPYR** | 0.017 | (Chuang et al. 1999) |  |  |  |  |  |  |  |  |
| **3-OH-BaPYR** | 0.0026 - 0.017 | (Chuang et al. 1999; Li et al. 2006) | 1.0 | (Campo et al. 2008, 2010) | 36 | (Campo et al. 2008) |  |  | 0.002 | (Khoury et al. 2018) |
| **7-OH-BaPYR** | 0.0026 | (Li et al. 2006) |  |  |  |  |  |  |  |  |
| **6-OH-IndPy** | 0.017 | (Chuang et al. 1999) |  |  |  |  |  |  |  |  |

Reference

Alghamdi, M. A., Alam, M. S., Stark, C., Mohammed, N., Harrison, R. M., Shamy, M., et al. (2015). Urinary metabolites of polycyclic aromatic hydrocarbons in Saudi Arabian schoolchildren in relation to sources of exposure. *Environmental Research*, *140*, 495–501. https://doi.org/10.1016/j.envres.2015.04.023

Alhamdow, A., Lindh, C., Albin, M., Gustavsson, P., Tinnerberg, H., & Broberg, K. (2017). Early markers of cardiovascular disease are associated with occupational exposure to polycyclic aromatic hydrocarbons. *Scientific Reports*, *7*(1), 1–11. https://doi.org/10.1038/s41598-017-09956-x

Barbeau, D., Maître, A., & Marques, M. (2011). Highly sensitive routine method for urinary 3-hydroxybenzo[a]pyrene quantitation using liquid chromatography-fluorescence detection and automated off-line solid phase extraction. *Analyst*, *136*(6), 1183–1191. https://doi.org/10.1039/c0an00428f

Becker, K., Kaus, S., Krause, C., Lepom, P., Schulz, C., Seiwert, M., & Seifert, B. (2002). German Environmental Survey 1998 (GerES III): Environmental pollutants in blood of the German population. *International Journal of Hygiene and Environmental Health*, *205*(4), 297–308. https://doi.org/10.1078/1438-4639-00155

Boogaard, P. J., & van Sittert, N. J. (1995). Urinary 1-hydroxypyrene as biomarker of exposure to polycyclic aromatic hydrocarbons in workers in petrochemical industries: baseline values and dermal uptake. *Science of the Total Environment*, *163*(1–3), 203–209. https://doi.org/10.1016/0048-9697(95)04481-F

Campo, L., Rossella, F., & Fustinoni, S. (2008). Development of a gas chromatography/mass spectrometry method to quantify several urinary monohydroxy metabolites of polycyclic aromatic hydrocarbons in occupationally exposed subjects. *Journal of Chromatography B: Analytical Technologies in the Biomedical and Life Sciences*, *875*(2), 531–540. https://doi.org/10.1016/j.jchromb.2008.10.017

Campo, L., Rossella, F., Pavanello, S., Mielzynska, D., Siwinska, E., Kapka, L., et al. (2010). Urinary profiles to assess polycyclic aromatic hydrocarbons exposure in coke-oven workers. *Toxicology Letters*, *192*(1), 72–78. https://doi.org/10.1016/J.TOXLET.2008.12.018

Cavanagh, J. A. E., Brown, L., Trought, K., Kingham, S., & Epton, M. J. (2007). Elevated concentrations of 1-hydroxypyrene in schoolchildren during winter in Christchurch, New Zealand. *Science of the Total Environment*, *374*(1), 51–59. https://doi.org/10.1016/j.scitotenv.2006.11.042

Chetiyanukornkul, T., Toriba, A., Kameda, T., Tang, N., & Hayakawa, K. (2006). Simultaneous determination of urinary hydroxylated metabolites of naphthalene, fluorene, phenanthrene, fluoranthene and pyrene as multiple biomarkers of exposure to polycyclic aromatic hydrocarbons. *Analytical and Bioanalytical Chemistry*, *386*(3), 712–718. https://doi.org/10.1007/s00216-006-0628-6

Chetiyanukornkul, T., Toriba, A., Kizu, R., & Hayakawa, K. (2004). Urinary 2-hydroxyfluorene and 1-hydroxypyrene levels in smokers and nonsmokers in Japan and Thailand. *Polycyclic Aromatic Compounds*, *24*(4–5), 467–474. https://doi.org/10.1080/10406630490471483

Choi, W., Kim, S., Baek, Y. W., Choi, K., Lee, K., Kim, S., et al. (2017). Exposure to environmental chemicals among Korean adults-updates from the second Korean National Environmental Health Survey (2012–2014). *International Journal of Hygiene and Environmental Health*, *220*(2), 29–35. https://doi.org/10.1016/j.ijheh.2016.10.002

Chuang, J. C., Callahan, P. J., Lyu, C. W., & Wilson, N. K. (1999). Polycyclic aromatic hydrocarbon exposures of children in low-income families. *Journal of Exposure Analysis and Environmental Epidemiology*, *9*(2), 85–98. https://doi.org/10.1038/sj.jea.7500003

de Oliveira Galvão, M. F., de Queiroz, J. D. F., Duarte, E. de S. F., Hoelzemann, J. J., de André, P. A., Saldiva, P. H. N., et al. (2017). Characterization of the particulate matter and relationship between buccal micronucleus and urinary 1-hydroxypyrene levels among cashew nut roasting workers. *Environmental Pollution*, *220*, 659–671. https://doi.org/10.1016/j.envpol.2016.10.024

Dobraca, D., Lum, R., Sjödin, A., Calafat, A. M., Laurent, C. A., Kushi, L. H., & Windham, G. C. (2018). Urinary biomarkers of polycyclic aromatic hydrocarbons in pre- and peri-pubertal girls in Northern California: Predictors of exposure and temporal variability. *Environmental Research*, *165*(October 2017), 46–54. https://doi.org/10.1016/j.envres.2017.11.011

Fiala, Z., Vyskocil, A., Krajak, V., Viau, C., Ettlerova, E., Bukac, J., et al. (2001). Environmental exposure of small children to polycyclic aromatic hydrocarbons. *International Archives of Occupational and Environmental Health*, *74*(6), 411–420. https://doi.org/10.1007/s004200100239

Freire, C., Abril, A., Fernández, M. F., Ramos, R., Estarlich, M., Manrique, A., et al. (2009). Urinary 1-hydroxypyrene and PAH exposure in 4-year-old Spanish children. *Science of the Total Environment*, *407*(5), 1562–1569. https://doi.org/10.1016/j.scitotenv.2008.10.068

Gilbert, N. L., & Viau, C. (1997). Biological monitoring of environmental exposure to PAHs in the vicinity of a Soderberg aluminium reduction plant. *Occupational and Environmental Medicine*, *54*(8), 619–621. https://doi.org/10.1136/oem.54.8.619

Göen T., Gündel J., Schaller K.-H., A. J. (1995a). The elimination of 1-hydroxypyrene in the urine of the general population and workers with different occupational exposures to PAH. *The Science of the Total Environment*, *163*, 195–201.

Göen T., Gündel J., Schaller K.-H., A. J. (1995b). The elimination of 1-hydroxypyrene in the urine of the general population and workers with different occupational exposures to PAH, *97*(95).

Grainger, J., Huang, W., Patterson, D. G., Turner, W. E., Pirkle, J., Caudill, S. P., et al. (2006). Reference range levels of polycyclic aromatic hydrocarbons in the US population by measurement of urinary monohydroxy metabolites. *Environmental Research*, *100*(3), 394–423. https://doi.org/10.1016/j.envres.2005.06.004

Guo, J., Huang, Y., Bian, S., Zhao, C., Jin, Y., Yu, D., et al. (2018). Associations of urinary polycyclic aromatic hydrocarbons with bone mass density and osteoporosis in U.S. adults, NHANES 2005–2010. *Environmental Pollution*, *240*, 209–218. https://doi.org/10.1016/j.envpol.2018.04.108

Guo, Y., Senthilkumar, K., Alomirah, H., Moon, H. B., Minh, T. B., Mohd, M. A., et al. (2013). Concentrations and profiles of urinary polycyclic aromatic hydrocarbon metabolites (OH-PAHs) in several Asian countries. *Environmental Science and Technology*, *47*(6), 2932–2938. https://doi.org/10.1021/es3052262

Hansen, Å. M., Raaschou-Nielsen, O., & Knudsen, L. E. (2006). Urinary 1-hydroxypyrene in children living in city and rural residences in Denmark. *Science of the Total Environment*, *363*(1–3), 70–77. https://doi.org/10.1016/j.scitotenv.2005.06.017

Hemat, H., Wittsiepe, J., Wilhelm, M., Müller, J., & Göen, T. (2012). High levels of 1-hydroxypyrene and hydroxyphenanthrenes in urine of children and adults from Afghanistan. *Journal of Exposure Science and Environmental Epidemiology*, *22*(1), 46–51. https://doi.org/10.1038/jes.2011.33

Heudorf, U., & Angerer, J. (2001a). Internal exposure to PAHs of children and adults living in homes with parquet flooring containing high levels of PAHs in the parquet glue. *International Archives of Occupational and Environmental Health*, *74*(2), 91–101. https://doi.org/10.1007/s004200000214

Heudorf, U., & Angerer, J. (2001b). Urinary monohydroxylated phenanthrenes and hydroxypyrene - The effects of smoking habits and changes induced by smoking on monooxygenase-mediated metabolism. *International Archives of Occupational and Environmental Health*, *74*(3), 177–183. https://doi.org/10.1007/s004200000215

Kang, J. W., Kim, H., Lee, C. H., & Cho, S. H. (2002). Correlation of Urinary 1-Hydroxypyrene and 2-Naphthol with Total Suspended Particulates in Ambient Air in Municipal Middle-School Students in Korea. *Archives of Environmental Health*, *57*(4), 377–382. https://doi.org/10.1080/00039890209601425

Karahalil, B., Burgaz, S., Fişek, G., & Karakaya, A. E. (1998). Biological monitoring of young workers exposed to polycyclic aromatic hydrocarbons in engine repair workshops. *Mutation Research - Genetic Toxicology and Environmental Mutagenesis*, *412*(3), 261–269. https://doi.org/10.1016/S1383-5718(97)00197-6

Khoury, C., Werry, K., Haines, D., Walker, M., & Malowany, M. (2018). Human biomonitoring reference values for some non-persistent chemicals in blood and urine derived from the Canadian Health Measures Survey 2009–2013. *International Journal of Hygiene and Environmental Health*, *221*(4), 684–696. https://doi.org/10.1016/j.ijheh.2018.03.003

Kuo, C. T., Chen, H. W., & Chen, J. L. (2004). Determination of 1-hydroxypyrene in children urine using column-switching liquid chromatography and fluorescence detection. *Journal of Chromatography B: Analytical Technologies in the Biomedical and Life Sciences*, *805*(2), 187–193. https://doi.org/10.1016/j.jchromb.2003.12.012

Lafontaine, M., Champmartin, C., Simon, P., Delsaut, P., & Funck-Brentano, C. (2006). 3-Hydroxybenzo[a]pyrene in the urine of smokers and non-smokers. *Toxicology Letters*, *162*(2-3 SPEC. ISS.), 181–185. https://doi.org/10.1016/j.toxlet.2005.09.019

Lee, C. Y., Lee, J. Y., Kang, J. W., & Kim, H. (2001). Effects of genetic polymorphisms of CYP1A1, CYP2E1, GSTM1, and GSTT1 on the urinary levels of 1-hydroxypyrene and 2-naphthol in aircraft maintenance workers. *Toxicology Letters*, *123*(2–3), 115–124. https://doi.org/10.1016/S0378-4274(01)00374-5

Leroyer, A., Jeandel, F., Maitre, A., Howsam, M., Deplanque, D., Mazzuca, M., & Nisse, C. (2010). 1-Hydroxypyrene and 3-hydroxybenzo[a]pyrene as biomarkers of exposure to PAH in various environmental exposure situations. *Science of the Total Environment*, *408*(5), 1166–1173. https://doi.org/10.1016/j.scitotenv.2009.10.073

Li, Z., Mulholland, J. A., Romanoff, L. C., Pittman, E. N., Trinidad, D. A., Lewin, M. D., & Sjödin, A. (2010). Assessment of non-occupational exposure to polycyclic aromatic hydrocarbons through personal air sampling and urinary biomonitoring. *Journal of Environmental Monitoring*, *12*(5), 1110–1118. https://doi.org/10.1039/c000689k

Li, Z., Romanoff, L., Bartell, S., Pittman, E. N., Trinidad, D. A., McClean, M., et al. (2012). Excretion Profiles and half-lives of ten urinary polycyclic aromatic hydrocarbon metabolites after dietary exposure. *Chemical Research in Toxicology*, *25*(7), 1452–1461. https://doi.org/10.1021/tx300108e

Li, Z., Romanoff, L. C., Trinidad, D. A., Hussain, N., Jones, R. S., Porter, E. N., et al. (2006). Measurement of urinary monohydroxy polycyclic aromatic hydrocarbons using automated liquid-liquid extraction and gas chromatography/isotope dilution high-resolution mass spectrometry. *Analytical Chemistry*, *78*(16), 5744–5751. https://doi.org/10.1021/ac0606094

Li, Z., Sandau, C. D., Romanoff, L. C., Caudill, S. P., Sjodin, A., Needham, L. L., & Patterson, D. G. (2008). bgb. *Environmental Research*, *107*(3), 320–331. https://doi.org/10.1016/j.envres.2008.01.013

Li, Z., Trinidad, D., Pittman, E. N., Riley, E. A., Sjodin, A., Dills, R. L., et al. (2016). Urinary polycyclic aromatic hydrocarbon metabolites as biomarkers to woodsmoke exposure-results from a controlled exposure study. *Journal of Exposure Science and Environmental Epidemiology*, *26*(3), 241–248. https://doi.org/10.1038/jes.2014.94

Liu, S., Liu, Q., Ostbye, T., Story, M., Deng, X., Chen, Y., et al. (2017). Levels and risk factors for urinary metabolites of polycyclic aromatic hydrocarbons in children living in Chongqing, China. *Science of the Total Environment*, *598*(1), 553–561. https://doi.org/10.1016/j.scitotenv.2017.04.103

Llop, S., Ballester, F., Estarlich, M., Ibarluzea, J., Manrique, A., Rebagliato, M., et al. (2008). Urinary 1-hydroxypyrene, air pollution exposure and associated life style factors in pregnant women. *Science of the Total Environment*, *407*(1), 97–104. https://doi.org/10.1016/j.scitotenv.2008.07.070

Martínez-Salinas, R. I., Elena Leal, M., Batres-Esquivel, L. E., Domínguez-Cortinas, G., Calderón, J., Díaz-Barriga, F., & Pérez-Maldonado, I. N. (2010). Exposure of children to polycyclic aromatic hydrocarbons in Mexico: Assessment of multiple sources. *International Archives of Occupational and Environmental Health*, *83*(6), 617–623. https://doi.org/10.1007/s00420-009-0482-x

McClean, M. D., Rinehart, R. D., Ngo, L., Eisen, E. A., Kelsey, K. T., Wiencke, J. K., & Herrick, R. F. (2004). Urinary 1-hydroxypyrene and polycyclic aromatic hydrocarbon exposure among asphalt paving workers. *Annals of Occupational Hygiene*, *48*(6), 565–578. https://doi.org/10.1093/annhyg/meh044

Meeker, J. D., Barr, D. B., Serdar, B., Rappaport, S. M., & Hauser, R. (2007). Utility of urinary 1-naphthol and 2-naphthol levels to assess environmental carbaryl and naphthalene exposure in an epidemiology study. *Journal of Exposure Science and Environmental Epidemiology*, *17*(4), 314–320. https://doi.org/10.1038/sj.jes.7500502

Mielzyńska, D., Siwińska, E., Kapka, L., Szyfter, K., Knudsen, L. E., & Merlo, D. F. (2006). The influence of environmental exposure to complex mixtures including PAHs and lead on genotoxic effects in children living in Upper Silesia, Poland. *Mutagenesis*, *21*(5), 295–304. https://doi.org/10.1093/mutage/gel037

Morgan, M. K., Jones, P. A., Sobus, J. R., Chuang, J. C., & Wilson, N. K. (2015). Using urinary biomarkers to evaluate polycyclic aromatic hydrocarbon exposure in 126 preschool children in Ohio. *International Journal of Environmental Health Research*, *25*(6), 628–639. https://doi.org/10.1080/09603123.2014.1003039

Mori, T., Yoshinaga, J., Suzuki, K., Mizoi, M., Adachi, S. ichi, Tao, H., et al. (2011). Exposure to polycyclic aromatic hydrocarbons, arsenic and environmental tobacco smoke, nutrient intake, and oxidative stress in Japanese preschool children. *Science of the Total Environment*, *409*(15), 2881–2887. https://doi.org/10.1016/j.scitotenv.2011.04.028

Mucha, A. P., Hryhorczuk, D., Serdyuk, A., Nakonechny, J., Zvinchuk, A., Erdal, S., et al. (2006). Urinary 1-hydroxypyrene as a biomarker of PAH exposure in 3-year-old Ukrainian children. *Environmental Health Perspectives*, *114*(4), 603–609. https://doi.org/10.1289/ehp.7898

Naksen, W., Kawichai, S., Srinual, N., Salrasee, W., & Prapamontol, T. (2017). First evidence of high urinary 1-hydroxypyrene level among rural school children during smoke haze episode in Chiang Mai Province, Thailand. *Atmospheric Pollution Research*, *8*(3), 418–427. https://doi.org/10.1016/j.apr.2016.11.002

Nethery, E., Wheeler, A. J., Fisher, M., Sjödin, A., Li, Z., Romanoff, L. C., et al. (2012). Urinary polycyclic aromatic hydrocarbons as a biomarker of exposure to PAHs in air: A pilot study among pregnant women. *Journal of Exposure Science and Environmental Epidemiology*, *22*(1), 70–81. https://doi.org/10.1038/jes.2011.32

Ochoa-Martinez, A. C., Orta-Garcia, S. T., Rico-Escobar, E. M., Carrizales-Yañez, L., Del Campo, J. D. M., Pruneda-Alvarez, L. G., et al. (2016). Exposure Assessment to Environmental Chemicals in Children from Ciudad Juarez, Chihuahua, Mexico. *Archives of Environmental Contamination and Toxicology*, *70*(4), 657–670. https://doi.org/10.1007/s00244-016-0273-9

Ochoa-Martínez, Á. C., Ruíz-Vera, T., Almendarez-Reyna, C. I., Orta-García, S. T., & Pérez-Maldonado, I. N. (2017). Influence on serum asymmetric dimethylarginine (ADMA) concentrations of human paraoxonase 1 polymorphism (Q192R) and exposure to polycyclic aromatic hydrocarbons (PAHs) in Mexican women, a gene-environment interaction. *Chemosphere*, *186*(550), 770–779. https://doi.org/10.1016/j.chemosphere.2017.08.055

Oliveira, M., Slezakova, K., Delerue-Matos, C., do Carmo Pereira, M., & Morais, S. (2016). Assessment of exposure to polycyclic aromatic hydrocarbons in preschool children: Levels and impact of preschool indoor air on excretion of main urinary monohydroxyl metabolites. *Journal of Hazardous Materials*, *322*, 357–369. https://doi.org/10.1016/j.jhazmat.2016.10.004

Preuss, R., Koch, H. M., Wilhelm, M., Pischetsrieder, M., & Angerer, J. (2004). Pilot study on the naphthalene exposure of German adults and children by means of urinary 1- and 2-naphthol levels. *International Journal of Hygiene and Environmental Health*, *207*(5), 441–445. https://doi.org/10.1078/1438-4639-00313

Ranjbar, M., Rotondi, M. A., Ardern, C. I., & Kuk, J. L. (2015). Urinary biomarkers of polycyclic aromatic hydrocarbons are associated with cardiometabolic health risk. *PLoS ONE*, *10*(9). https://doi.org/10.1371/journal.pone.0137536

Rodrigues, E. G., Smith, K., Maule, A. L., Sjodin, A., Li, Z., Romanoff, L., et al. (2014). Urinary polycyclic aromatic hydrocarbon (OH-PAH) metabolite concentrations and the effect of GST polymorphisms among US Air force personnel exposed to jet fuel. *Journal of Occupational and Environmental Medicine*, *56*(5), 465–471. https://doi.org/10.1097/JOM.0000000000000142

Schulz, C., Becker, K., & Seiwert, M. (2008). German Environmental Survey for children 2003/06 - GerES IV - Human Biomonitoring Levels of selected substances in blood and urine of children in Germany. *Gesundheitswesen*, *64*(SUPPL. 1). https://doi.org/10.1055/s-2002-39224

Shahsavani, S., Dehghani, M., Hoseini, M., & Fararouei, M. (2017). Biological monitoring of urinary 1-hydroxypyrene by PAHs exposure among primary school students in Shiraz, Iran. *International Archives of Occupational and Environmental Health*, *90*(2), 179–187. https://doi.org/10.1007/s00420-016-1184-9

Siwińska, E., Mielzyńska, D., Smolik, E., Bubak, A., & Kwapuliński, J. (1998). Evaluation of intra- and interindividual variation of urinary 1- hydroxypyrene, a biomarker of exposure to polycyclic aromatic hydrocarbons. *Science of the Total Environment*, *217*(1–2), 175–183. https://doi.org/10.1016/S0048-9697(98)00186-7

Siwińska, Ewa, Mielzyńska, D., Bubak, A., & Smolik, E. (1999). The effect of coal stoves and environmental tobacco smoke on the level of urinary 1-hydroxypyrene. *Mutation Research - Genetic Toxicology and Environmental Mutagenesis*, *445*(2), 147–153. https://doi.org/10.1016/S1383-5718(99)00121-7

Sochacka-Tatara, E., Majewska, R., Perera, F. P., Camann, D., Spengler, J., Wheelock, K., et al. (2018). Urinary polycyclic aromatic hydrocarbon metabolites among 3-year-old children from Krakow, Poland. *Environmental Research*, *164*(March), 212–220. https://doi.org/10.1016/j.envres.2018.02.032

Sul, D., Ahn, R., Im, H., Oh, E., Kim, J. H., Kim, J. G., et al. (2012). Korea National Survey for Environmental Pollutants in the human body 2008: 1-hydroxypyrene, 2-naphthol, and cotinine in urine of the Korean population. *Environmental Research*, *118*, 25–30. https://doi.org/10.1016/j.envres.2012.07.010

Thai, P. K., Heffernan, A. L., Toms, L. M. L., Li, Z., Calafat, A. M., Hobson, P., et al. (2016). Monitoring exposure to polycyclic aromatic hydrocarbons in an Australian population using pooled urine samples. *Environment International*, *88*, 30–35. https://doi.org/10.1016/j.envint.2015.11.019

Tsai, H.-T., Wu, M.-T., Hauser, R., Rodrigues, E., Ho, C.-K., Liu, C.-L., & Christiani, D. C. (2003). Exposure to environmental tobacco smoke and urinary 1-hydroxypyrene levels in preschool children. *The Kaohsiung Journal of Medical Sciences*, *19*(3), 97–104. https://doi.org/10.1016/S1607-551X(09)70456-5

Väänänen, V., Hämeilä, M., Kontsas, H., Peltonen, K., & Heikkilä, P. (2003). Air concentrations and urinary metabolites of polycyclic aromatic hydrocarbons among paving and remixing workers. *Journal of Environmental Monitoring*, *5*(5), 739–746. https://doi.org/10.1039/b304096h

Van Delft, J. H. M., Steenwinkel, M. J. S. T., Van Asten, J. G., De Vogel, N., Bruijntjes-Rozier, T. C. D. M., Schouten, T., et al. (2001). Biological monitoring the exposure to polycyclic aromatic hydrocarbons of coke oven workers in relation to smoking and genetic polymorphisms for GSTM1 and GSTT1. *Annals of Occupational Hygiene*, *45*(5), 395–408. https://doi.org/10.1016/S0003-4878(00)00065-X

Van Wijnen, J. H., Slob, R., Jongmans-Liedekerken, G., Van De Weerdt, R. H. J., & Woudenberg, F. (1996). Exposure to polycyclic aromatic hydrocarbons among Dutch children. *Environmental Health Perspectives*, *104*(5), 530–534. https://doi.org/10.1289/ehp.96104530

Viau, C., Vyskočil, A., & Martel, L. (1995). Background urinary 1-hydroxypyrene levels in non-occupationally exposed individuals in the Province of Québec, Canada, and comparison with its excretion in workers exposed to PAH mixtures. *Science of the Total Environment*, *163*(1–3), 191–194. https://doi.org/10.1016/0048-9697(95)04496-N

Wang, Y., Meng, L., Pittman, E. N., Etheredge, A., Hubbard, K., Trinidad, D. A., et al. (2017). Quantification of urinary mono-hydroxylated metabolites of polycyclic aromatic hydrocarbons by on-line solid phase extraction-high performance liquid chromatography-tandem mass spectrometry. *Analytical and Bioanalytical Chemistry*, *409*(4), 931–937. https://doi.org/10.1007/s00216-016-9933-x

Xia, Y., Zhu, P., Han, Y., Lu, C., Wang, S., Gu, A., et al. (2009). Urinary metabolites of polycyclic aromatic hydrocarbons in relation to idiopathic male infertility. *Human Reproduction*, *24*(5), 1067–1074. https://doi.org/10.1093/humrep/dep006

Xu, Xiaohui, Cook, R. L., Ilacqua, V. A., Kan, H., Talbott, E. O., & Kearney, G. (2010). Studying associations between urinary metabolites of polycyclic aromatic hydrocarbons (PAHs) and cardiovascular diseases in the United States. *Science of the Total Environment*, *408*(21), 4943–4948. https://doi.org/10.1016/j.scitotenv.2010.07.034

Xu, Xu, Zhang, J., Zhang, L., Liu, W., & Weisel, C. P. (2004). Selective detection of monohydroxy metabolites of polycyclic aromatic hydrocarbons in urine using liquid chromatography/triple quadrupole tandem mass spectrometry. *Rapid Communications in Mass Spectrometry*, *18*(19), 2299–2308. https://doi.org/10.1002/rcm.1625

Yang, J., Liu, Y., Zhang, H., Zhang, H., Wang, W., & Fan, Y. (2018). Urinary 1-hydroxypyrene and smoking are determinants of LINE-1 and AhRR promoter methylation in coke oven workers. *Mutation Research - Genetic Toxicology and Environmental Mutagenesis*, *826*(January), 33–40. https://doi.org/10.1016/j.mrgentox.2018.01.001

Yang, L., Zhou, Y., Sun, H., Lai, H., Liu, C., Yan, K., et al. (2014). Dose-response relationship between polycyclic aromatic hydrocarbon metabolites and risk of diabetes in the general Chinese population. *Environmental Pollution*, *195*, 24–30. https://doi.org/10.1016/j.envpol.2014.08.012

Yoon, H. S., Lee, K. M., Lee, K. H., Kim, S., Choi, K., & Kang, D. (2012). Polycyclic aromatic hydrocarbon (1-OHPG and 2-naphthol) and oxidative stress (malondialdehyde) biomarkers in urine among Korean adults and children. *International Journal of Hygiene and Environmental Health*, *215*(4), 458–464. https://doi.org/10.1016/j.ijheh.2012.02.007

Zhu, P., Bian, Z., Xia, Y., Han, Y., Qiao, S., Zhao, R., et al. (2009). Relationship between urinary metabolites of polycyclic aromatic hydrocarbons and thyroid hormone levels in Chinese non-occupational exposure adult males. *Chemosphere*, *77*(7), 883–888. https://doi.org/10.1016/j.chemosphere.2009.08.054
